# Screening Strategies to Reduce COVID-19 Mortality in Nursing Homes

**DOI:** 10.1001/jamahealthforum.2024.0688

**Published:** 2024-04-26

**Authors:** Shirley Dong, Eric Jutkowitz, John Giardina, Alyssa Bilinski

**Affiliations:** 1 Department of Health Services, Policy & Practice, Brown University School of Public Health, Providence, Rhode Island; 2 Center of Innovation in Long Term Services and Supports, Providence VA Medical Center, Providence, Rhode Island; 3 Evidence Synthesis Program Center Providence VA Medical Center, Providence, Rhode Island; 4 Medical Practice Evaluation Center, Massachusetts General Hospital, Boston; 5 Department of Biostatistics, Brown University School of Public Health, Providence, Rhode Island

## Abstract

**Importance:**

Nursing home residents continue to bear a disproportionate share of COVID-19 morbidity and mortality, accounting for 9% of all US COVID-19 deaths in 2023, despite comprising only 0.4% of the population.

**Objective:**

To evaluate the cost-effectiveness of screening strategies in reducing COVID-19 mortality in nursing homes.

**Design and Setting:**

An agent-based model was developed to simulate SARS-CoV-2 transmission in the nursing home setting. Parameters were determined using SARS-CoV-2 virus data and COVID-19 data from the Centers for Medicare & Medicaid Services and US Centers for Disease Control and Prevention that were published between 2020 and 2023, as well as data on nursing homes published between 2010 and 2023. The model used in this study simulated interactions and SARS-CoV-2 transmission between residents, staff, and visitors in a nursing home setting. The population used in the simulation model was based on the size of the average US nursing home and recommended staffing levels, with 90 residents, 90 visitors (1 per resident), and 83 nursing staff members.

**Exposure:**

Screening frequency (none, weekly, and twice weekly) was varied over 30 days against varying levels of COVID-19 community incidence, booster uptake, and antiviral use.

**Main Outcomes and Measures:**

The main outcomes were SARS-CoV-2 infections, detected cases per 1000 tests, and incremental cost of screening per life-year gained.

**Results:**

Nursing home interactions were modeled between 90 residents, 90 visitors, and 83 nursing staff over 30 days, completing 4000 to 8000 simulations per parameter combination. The incremental cost-effectiveness ratios of weekly and twice-weekly screening were less than $150 000 per resident life-year with moderate (50 cases per 100 000) and high (100 cases per 100 000) COVID-19 community incidence across low–booster uptake and high–booster uptake levels. When COVID-19 antiviral use reached 100%, screening incremental cost-effectiveness ratios increased to more than $150 000 per life-year when booster uptake was low and community incidence was high.

**Conclusions and Relevance:**

The results of this cost-effectiveness analysis suggest that screening may be effective for reducing COVID-19 mortality in nursing homes when COVID-19 community incidence is high and/or booster uptake is low. Nursing home administrators can use these findings to guide planning in the context of widely varying levels of SARS-CoV-2 transmission and intervention measures across the US.

## Key Points

**Question:**

What is the cost-effectiveness of regular screening in terms of reducing COVID-19 mortality in nursing home residents?

**Findings:**

In this cost-effectiveness analysis, the simulation model projected that incremental cost-effectiveness ratios of weekly and twice-weekly screening were less than $150 000 per year of life with moderate (50 cases per 100 000) and high (100 cases per 100 000) COVID-19 community incidence across both low–booster uptake and high–booster uptake levels.

**Meaning:**

Screening may be a cost-effective approach to reducing COVID-19 mortality in nursing homes when COVID-19 community incidence is high and/or booster uptake is low.

## Teaser

This cost-effectiveness analysis examines screening strategies and their predicted association with reducing COVID-19 mortality in nursing homes.

## Introduction

Even as COVID-19 mortality has declined in the US since the height of the Omicron wave in January 2022,^1^ nursing home residents have continued to experience substantial rates of COVID-19 morbidity and mortality. COVID-19 remained the fourth leading cause of death in 2022,^2^ and from January 2023 to April 2023, nursing home residents accounted for more than 9% of COVID-19 deaths,^3,4^ despite comprising only 0.4% of the population.^5^ Nursing home residents are especially vulnerable to contracting SARS-CoV-2 because they live in close quarters and have frequent, close contact with staff and visitors. Residents are also susceptible to severe COVID-19 outcomes because most have multiple comorbidities.^6,7^

Nursing homes have adopted multipronged approaches to managing SARS-CoV-2 spread, including social distancing,^8^ air purification,^9^ masking,^10^ vaccination,^11^ testing,^12^ and antiviral treatments.^13^ Screening entails testing asymptomatic individuals, regardless of known exposure, to identify carriers of the virus and prevent severe outbreaks.^14^ This strategy has been shown to be an effective method in reducing COVID-19 cases and deaths in nursing home residents^12,15^ and can be scaled up or down based on the circumstances of an individual nursing home. However, whether to screen and when to increase or decrease screening frequency can be unclear. Currently, the US Centers for Disease Control and Prevention (CDC) guidelines require SARS-CoV-2 testing only when an individual displays symptoms of COVID-19 or is exposed to someone with a SARS-CoV-2 infection, and regular screening is at the discretion of individual nursing home facilities.^16^

In this cost-effectiveness analysis, we use an agent-based model to simulate SARS-CoV-2 transmission in nursing homes. We aimed to evaluate the cost-effectiveness of screening strategies that nursing home administrators can implement to reduce resident COVID-19 mortality. Previous nursing home SARS-CoV-2 modeling studies have focused on the period before vaccine availability and up to the emergence of the Delta variant.^17,18,19^ We address gaps in the literature by evaluating screening strategies in the context of an endemic Omicron variant and varying levels of community transmission, booster uptake, and antiviral use.

## Methods

### Model Structure

**Figure 1.**
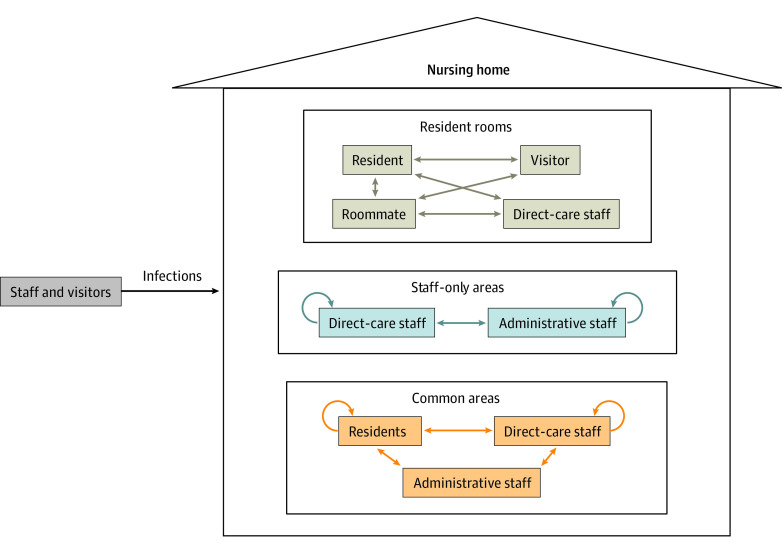
Model Schematic SARS-CoV-2 transmission was simulated in a nursing home setting by modeling interactions between residents, staff, and visitors. The black arrow represents SARS-CoV-2 brought into the nursing home by staff and visitors. The daily probability of SARS-CoV-2 infection outside the nursing home depends on the community incidence (number of cases per 100 000 population per day). Within a nursing home, transmission can occur in resident rooms, staff-only areas, and common areas. Beige arrows represent transmission in a resident’s room. Blue arrows represent transmission between staff. Orange arrows represent transmission in shared spaces of the nursing home.

We developed an agent-based, susceptible-exposed-infectious-recovered model to project SARS-CoV-2 transmission in a nursing home (Figure 1). The model simulates interactions over 30 days between nursing home residents, staff, and visitors. Only staff and visitors import infections into the nursing home, yet residents, staff, and visitors can all transmit SARS-CoV-2 within the nursing home setting. SARS-CoV-2 transmission between agents can occur in resident rooms, common areas, and staff-only areas. The model simulates 8-hour increments. We built a synthetic population of 90 nursing home residents, 83 members of staff, and 90 visitors using an average national resident census count from 2019^20^ and recommended staffing levels.^21^

In the nursing home, the nursing staff roles include registered nurses, licensed practical nurses, certified nurse aides, and medication aides.^22,23^ We denote these staff members as direct care staff. There are also administrative staff who work in the nursing home but do not directly treat residents.^24^ Additional model parameters are detailed in Table 1.^4,16,18,19,20,21,25,26,27,28,29,30,31,32,33,34,35,36,37,38,39,40,41,42,43,44,45^

**Table 1.** Model Parameters

Model parameters	Value	Source
Nursing home parameters		
No. of residents	90	Centers for Medicare & Medicaid Services data^20^
No. of staff	83	Harrington et al,^21^ 2020
No. of visitors	90	Assumed 1 visitor per resident
Proportion of residents in shared rooms	0.74	LTCFocus^25^
No. of residents that a resident or staff member contacts in common areas per 8-h period	3	Assumed 50% increase from estimates used in Kahn et al,^18^ 2022, and Holmdahl et al,^19^ 2022, to reflect removal of social distancing restrictions (Centers for Medicare & Medicaid Services^26^)
No. of staff that a resident or staff member contacts in common areas per 8-h period	3	Assumed 50% increase from estimates used in Kahn et al,^18^ 2022, and Holmdahl et al,^19^ 2022, to reflect removal of social distancing restrictions (Centers for Medicare & Medicaid Services^26^)
No. of staff-to-staff member contacts in staff-only areas per 8-h period	6	Assumed 200% increase from estimates used in Kahn et al,^18^ 2022, and Holmdahl et al,^19^ 2022, to reflect removal of social distancing restrictions (Centers for Medicare & Medicaid Services^26^)
SARS-CoV-2 and COVID-19 parameters		
Daily attack rate of Omicron variant (attack rate in resident rooms)	0.18	Baker et al,^27^ 2022
Relative attack rate in common areas compared with resident rooms	0.25	Assumed 2 h of each 8-h period are spent in common areas
Relative attack rate among staff compared with resident rooms	0.25	Assumed 2 h of each 8-h period are spent in staff-only areas
Proportion of the nursing home that contracts SARS-CoV-2 is asymptomatic	0.50	Ma et al,^28^ 2021; Joung et al,^29^ 2022
Community incidence, cases per 100 000 population (low, moderate, high)	(5, 50, 100)	*New York Times*^30^; Centers for Disease Control and Prevention^31^
Latent period, d^a^	γ(4.45, 1.42)	Xin et al,^32^ 2022
Incubation period, d^b^	γ(8.38, 2.20)	Xin et al,^32^ 2022
Length of infectious period, d	5	Centers for Disease Control and Prevention^33^
Length of self-isolation at place of residence on COVID-19 diagnosis (residents, staff, visitors), d^c^	(10, 7, 5)	Centers for Disease Control and Prevention^16,34,35^
Case-to-fatality ratio	0.018	Centers for Medicare & Medicaid Services data^4^
Masking, vaccination, and antiviral parameters		
Reduction in SARS-CoV-2 transmission due to masking	0.70	Centers for Disease Control and Prevention^36^
Vaccine efficacy (primary series/previous infection, booster dose)	(0.40, 0.70)	Chin et al,^37^ 2022
Low booster uptake proportion (residents, staff, visitors)	(0.48, 0.22, 0.07)	Centers for Medicare & Medicaid Services Data^4^; Centers for Disease Control and Prevention^38^
High booster uptake proportion (residents, staff, visitors)	(0.74, 0.51, 0.34)	Chidambaram et al,^39^ 2022; *New York Times*^40^
Antiviral treatment effectiveness against death	0.71	Dryden-Peterson et al,^41^ 2022
Testing parameters		
Test sensitivity	0.84	Pollock et al,^42^ 2021; Schrom et al,^43^ 2022
Proportion of nursing home residents and staff tested	0.90	Assumed using baseline test uptake proportion in Bilinski et al,^44^ 2021; Giardina et al,^45^ 2022

^a^ The latent period of infection is the length of time between exposure to the virus and the start of infectiousness.

^b^ The incubation period is the length of time between exposure to the virus and the appearance of symptoms (if symptomatic).

^c^ Residents isolate for 10 days in the nursing home, staff members isolate for 7 days at home, and visitors isolate for 5 days at home.

The study follows the Consolidated Health Economic Evaluation Reporting Standards (CHEERS) reporting guideline^46^ and was deemed exempt from institutional review board approval and informed consent using Brown University’s Human Subjects Research Self-Determination Tool.^47^

### Importation of SARS-CoV-2

Staff and visitors import SARS-CoV-2 into the nursing home from the community. The daily probability of importing SARS-CoV-2 into the nursing home is based on the number of COVID-19 cases in the community per 100 000 population per day. Observed COVID-19 community incidence varies from 5 to 100 cases per 100 000 population per day. To account for the underreporting of SARS-CoV-2 transmission, we multiplied the community incidence range by 10 when simulating strategies so that the true community incidence ranged from 50 to 1000 infections per 100 000 population per day.^48^ For context, the average daily reported community incidence during the Omicron wave from December 2021 to February 2022 was 100 cases per 100 000 population per day,^31^ so the maximum community incidence roughly corresponds to an Omicron-type wave, while accounting for underreporting.

### Contacts and Schedule

We simulated 8-hour shifts across 30 days, assuming staff rotated 3 times in 24 hours. Each staff member is assigned a morning, evening, or night shift. Residents do not leave the nursing home and are present during all shifts.

#### Resident Rooms

In the simulation model, a resident has contact with 11 direct care staff members per day. Residents also have contact with a visitor in their rooms during morning shifts 4 to 5 times within 30 days, and visits occur on random days.

We assumed that Medicare/Medicaid payers have shared rooms, and residents paying out of pocket have private rooms. On average, 74% of residents’ nursing home stays are covered by Medicare/Medicaid.^25^ As such, in the synthetic cohort, 66 residents have shared rooms, and 24 residents have private rooms. Residents in shared rooms can transmit SARS-CoV-2 to their roommates. Visitors can transmit SARS-CoV-2 to both residents in a shared room.

#### Communal Areas

In common areas, each resident is assumed to have contact with 3 other residents and 3 staff members during each 8-hour shift. Each staff member is assumed to have contact with 3 residents and 3 other staff members during their assigned 8-hour shift. In staff-only areas, each staff member is assumed to contact 6 other staff members daily during their assigned shift. Contacts in common and staff-only areas include both direct care and administrative staff.

### SARS-CoV-2 Transmission

SARS-CoV-2 can be transmitted in resident rooms, staff-only areas, and common areas (Figure 1). The probability of a susceptible person getting infected with SARS-CoV-2 during each interaction with an infected person is a function of the susceptible person’s level of immunity, the attack rate of the virus, and the infected person’s use of masking. A susceptible person’s level of immunity is determined by vaccination or previous infection. Technical details on calculating the infection probability are explained in eMethods 1 in Supplement 1. We assume that only staff members in the nursing home are masked. We also assume that staff members adhere to masking properly and as much as possible such that if infected, their transmission risk is reduced by 0.7.^36^

### Diagnosis and Isolation

Individuals are diagnosed with a SARS-CoV-2 infection through the appearance of symptoms and/or a positive rapid antigen test when screened, whichever comes first. In the simulation model, we use CDC guidelines for the length of isolation when SARS-CoV-2 infection is diagnosed. Infected residents isolate in their rooms on average for 10 days^16^ and do not enter the common areas, while infected staff and visitors isolate at home for 7 days^34^ and 5 days,^35^ respectively.

### Vaccination and Antiviral Treatments

We used the national average vaccination rates for monovalent and bivalent booster shots as of December 2022 to denote low and high booster uptake, respectively (Table 1).^4,38,39,40^ See eMethods 2 in Supplement 1 for details on vaccination parameters.

Nirmatrelvir/ritonavir and molnupiravir are oral antiviral treatments authorized for use against symptomatic SARS-CoV-2 infection and are used particularly in those at high risk for hospitalization and death.^49^ We used the average rate of nirmatrelvir/ritonavir and molnupiravir uptake across all US nursing homes from January 2023 to April 2023 (32%)^4^ as the baseline uptake rate of antiviral use. We varied both vaccination and antiviral uptake in sensitivity analyses.

### Screening Strategies

We examined the morbidity and mortality outcomes over 30 days of (1) no screening, (2) weekly screening, and (3) twice-weekly screening of residents and staff members. We assumed that staff members who have positive results of a rapid antigen test return home and do not contribute to transmission in the nursing home. Visitors are not screened but do not enter the nursing home if they are experiencing symptoms. Individuals who have positive test results continue to be screened for the remainder of the 30 days, so the estimates of testing costs are conservative.

### Implementation and Outcomes

We projected both nursing home–acquired SARS-CoV-2 infections and detected cases over 30 days. Cases are defined as SARS-CoV-2 infections detected by testing; therefore, infections are higher than detected cases. We compared the number of nursing home–acquired infections by screening strategy (no screening, weekly screening, twice-weekly screening) and booster uptake (low, high). We conducted 4000 model simulations for each parameter combination, increasing to 8000 simulations for low–community incidence and high–booster uptake scenarios. We estimated the number of detected cases per 1000 tests for each strategy. Finally, we evaluated the incremental cost of screening per resident life-year gained by screening strategy and booster uptake level; the calculations for the cost of screening and the incremental cost-effectiveness ratio (ICER) are detailed in eMethods 1 in Supplement 1. We incorporate the cost of staff time to administer tests to residents as a factor in screening costs. In evaluating the ICER, we set a benchmark of cost-effectiveness at $150 000 per resident life-year gained.^50,51^ We assumed a life expectancy of approximately 1 year for residents^52^ and varied this assumption in sensitivity analyses. This model was implemented in R statistical software, version 4.2.2 (R Project for Statistical Computing), and the code can be viewed online.^53^

### Sensitivity Analysis

With public sentiment moving away from restrictive COVID-19 practices, we look at boosters and antiviral treatments as important tools in preventing COVID-19 deaths among nursing home residents. We examined the incremental cost of screening per resident life-year gained at varying levels of booster uptake, antiviral uptake, and masking adherence. Given the variation in rapid antigen test sensitivity based on an individual’s viral load at the time of testing and the brand of test used,^54^ we lowered the estimate of test sensitivity to 0.65 in another sensitivity analysis. We also varied the number of resident and staff contacts in communal areas by one-third and examined the scenario in which only staff are screened.

## Results

### Infections and Deaths in Residents and Staff

**Figure 2.**
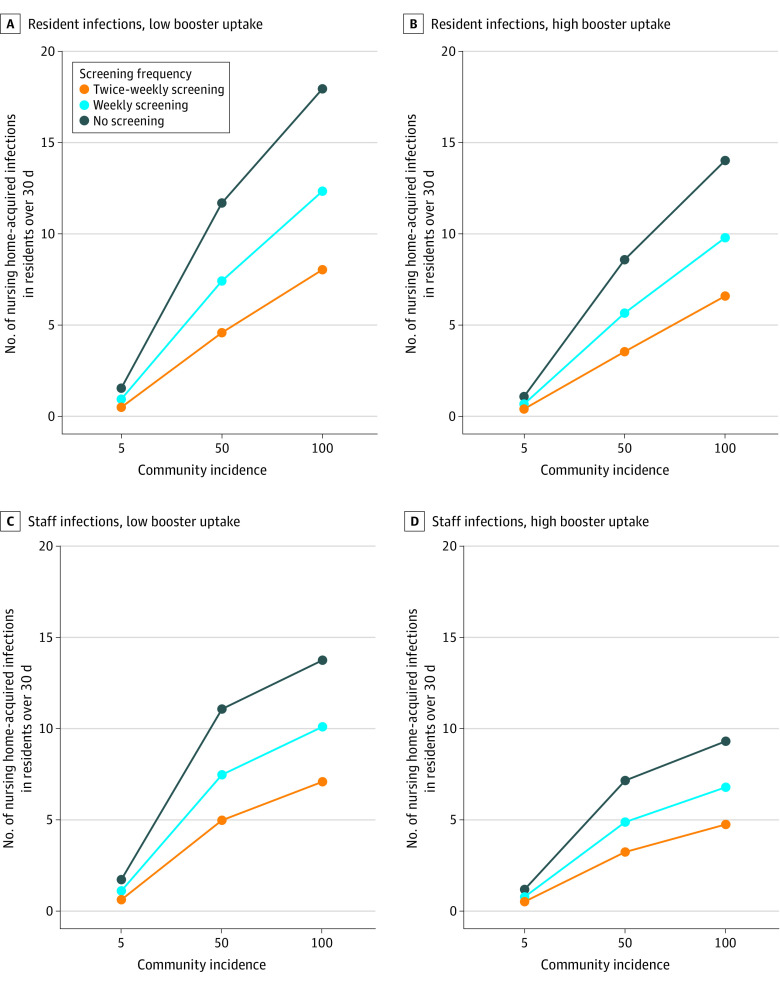
Number of Nursing-Home–Acquired Infections in Residents and Staff Panels A and B show the association of nursing home–acquired infections in residents across low and high booster uptake with community incidence by screening frequency. Panels C and D illustrate the association of nursing home–acquired infections in staff members across low and high booster uptake with community incidence by screening frequency. Community incidence is defined as the number of cases per 100 000 population per day. Infections include both detected and undetected infections. Low booster uptake was 48%, 22%, and 7% booster uptake among residents, staff, and visitors, respectively. High booster uptake was 74%, 51%, and 34% uptake among residents, staff, and visitors, respectively.

Under low booster uptake and no screening, monthly nursing home–acquired resident infections ranged from an average of 1736 infections per 100 000 residents at low community incidence (5 cases per 100 000) to 19 977 infections per 100 000 residents at high community incidence (100 cases per 100 000) (Figure 2). Nursing home–acquired staff infections ranged from 2108 to 16 606 infections per 100 000 staff at low to high community incidence. Weekly screening reduced the number of infections by 60% to 70% to 1065 to 13 739 infections per 100 000 residents and 1365 to 12 206 infections per 100 000 staff members. Twice-weekly screening further reduced the number of infections by another 55% to 65%, to 571 to 8955 infections per 100 000 residents, and 782 to 8575 infections per 100 000 staff. Without screening, resident deaths ranged from 31 to 360 deaths per 100 000 residents at low to high community incidence. Weekly screening reduced the number of deaths to range from 19 to 247 deaths per 100 000 residents, and twice-weekly screening further reduced deaths to range from 10 to 161 per 100 000 residents.

With high booster uptake, infections were lower across scenarios by an average factor of 0.75. Without screening, average monthly nursing home–acquired resident infections ranged from 1238 to 15 622 per 100 000 residents (low to high community incidence), and deaths ranged from 22 to 281 per 100 000 residents. Weekly screening under high booster uptake reduced the number of deaths to range from 14 to 197 per 100 000 residents, and twice-weekly screening reduced deaths to 9 to 133 per 100 000 residents.

### Detected Cases per 1000 Tests

At low booster uptake and low community incidence, the model predicted 1.3 and 1.4 monthly detected cases (2.0 and 1.1 detected cases per 1000 tests) under weekly and twice-weekly screening, respectively (Table 2). At high community incidence, the model predicted 17 and 23 detected cases (28 and 19 detected cases per 1000 tests) under weekly and twice-weekly screening.

**Table 2.** Detected Cases and Incremental Cost of Screening by Community Incidence and Booster Uptake^a^

Community incidence	Low booster uptake						High booster uptake						
	Weekly screening			Twice-weekly screening			Weekly screening			Twice-weekly screening			
**Low: 5 cases per 100 000 population**													
Tests	600			1200			600				1200		
Detected cases	1.3			1.4			1.0				1.3		
Detected cases per 1000 tests	2.0			1.1			1.6				1.0		
**Moderate: 50 cases per 100 000 population**													
Tests	600			1200			600				1200		
Detected cases	10			13			8.5				11		
Detected cases per 1000 tests	16			10			14				8.9		
**High: 100 cases per 100 000 population**													
Tests	600			1200			600				1200		
Detected cases	17			23			15				21		
Detected cases per 1000 tests	28			19			25				17		
**Strategy**	**Cost, $**	**Incremental cost, $**	**Resident deaths**		**Incremental reduction in deaths**	**Incremental cost per resident life-year gained, $**	**Cost, $**	**Incremental cost, $**	**Resident deaths**			**Incremental reduction in deaths**	**Incremental cost per resident life-year gained, $**
**Low: 5 cases per 100 000 population**													
No screening	0	NA	0.028		NA	NA	0	NA	0.020			NA	NA
Weekly screening	4000	4000	0.017		0.011	379 000	4000	4000	0.013			0.0074	557 000
Twice-weekly screening	8000	4000	0.0092		0.0080	513 000	8000	4000	0.0078			0.0049	841 000
**Moderate: 50 cases per 100 000 population**													
No screening	0	NA	0.21		NA	NA	0	NA	0.16			NA	NA
Weekly screening	4000	4000	0.13		0.077	53 000	4000	4000	0.10			0.053	78 000
Twice-weekly screening	8000	4000	0.083		0.051	80 000	8000	4000	0.064			0.038	107 000
**High: 100 cases per 100 000 population**													
No screening	0	NA	0.32		NA	NA	0	NA	0.25			NA	NA
Weekly screening	4000	4000	0.22		0.10	40 000	4000	4000	0.18			0.076	54 000
Twice-weekly screening	8000	4000	0.15		0.078	52 000	8000	4000	0.12			0.058	71 000

Abbreviation: NA, not applicable.

^a^ Tests are rounded to the nearest hundred, cases are rounded to 2 significant digits, detected cases per 1000 tests are rounded to 2 significant digits, costs are rounded to the nearest thousand, and deaths are rounded to 2 significant digits. Detected cases include only those infections that are detected to be SARS-CoV-2 using screening. The numbers presented in this table were obtained with calculations using exact values, not the rounded values presented in the table.

With high booster uptake and low community incidence, the model predicted 1.0 and 1.3 monthly detected cases (1.6 and 1.0 detected cases per 1000 tests) under weekly and twice-weekly screening, respectively. At high community incidence, the model predicted 15 and 21 detected cases (25 and 17 detected cases per 1000 tests) under weekly and twice-weekly screening.

In both low– and high–booster uptake scenarios, yield increased superlinearly with community incidence. The number of detected cases per 1000 tests under moderate community incidence was approximately 8 to 10 times more than under low community incidence. The number of detected cases per 1000 tests under high community incidence was approximately 2 times more than under moderate community incidence, and approximately 14 to 17 times more than under low community incidence.

The number of detected cases per 1000 tests was consistently higher under weekly screening than twice-weekly screening. However, this does not imply using fewer tests yields better detection; rather, the value of any single test in detecting infections goes down when a greater number of tests are used. This can be seen in Table 2, in which the number of detected cases under weekly screening is consistently lower than twice-weekly screening, but the number of detected cases per 1000 tests under weekly screening is consistently greater than twice-weekly screening.

### Incremental Cost-Effectiveness of Screening Strategies

Weekly screening of residents and staff in the nursing home over 30 days costs approximately $4000. Twice-weekly screening doubles that cost to approximately $8000. When community incidence was low (5 cases per 100 000), ICERs associated with weekly screening ranged from $379 000 under low booster uptake to $557 000 under high booster uptake (Table 2). ICERs associated with twice-weekly screening ranged from $513 000 to $841 000. However, with moderate (50 cases per 100 000) or high community incidence (100 cases per 100 000), ICERs fell to less than $80 000 across booster assumptions for weekly screening and less than $110 000 for twice-weekly screening.

### Sensitivity Analysis

**Figure 3.**
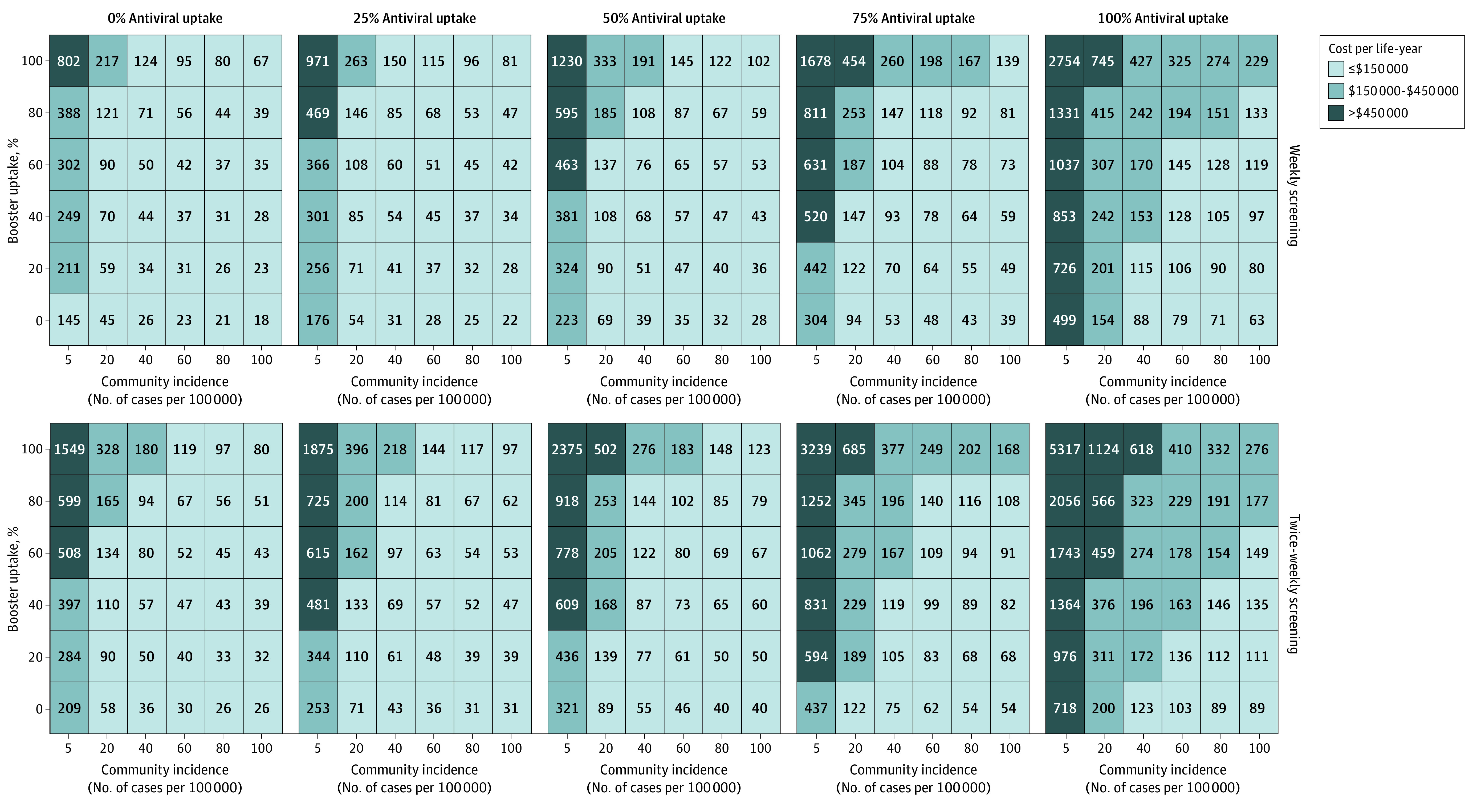
Incremental Cost of Screening per Resident Life-Year Gained Varied by Antiviral Treatment Uptake Costs are denoted in thousands of dollars and are rounded to the nearest thousand. At 0% booster uptake, the proportion of residents, staff, and visitors who received boosters is all 0%. At all other levels of booster uptake, the proportion of residents who received boosters is shown on the y-axis, the proportion of staff with boosters is half that of residents with boosters, and the proportion of visitors with boosters is a quarter that of residents with boosters (eg, at 20% booster uptake, the proportion of boosted residents, staff, and visitors is 20%, 10%, and 5%, respectively). These proportions are estimates made to reflect the relative proportions of booster uptake observed between resident, staff, and visitor populations. Those who have not been boosted are still assumed to have some immunity protection from the initial 2-dose vaccine series or previous infection.

Across all levels of booster and antiviral uptake, both weekly and twice-weekly screening ICERs generally rose to more than $150 000 per life-year at low community incidence (Figure 3). At 0% antiviral uptake, screening ICERs generally fell to less than $150 000 per life-year, provided that community incidence exceeded 5 cases per 100 000. Thresholds increased with higher antiviral usage. At 100% antiviral uptake, screening ICERs generally fell to less than $150 000 per life-year, only when booster uptake fell to less than 50%, and community incidence rose to more than 50 cases per 100 000.

The results were sensitive to estimates of resident life expectancy. When resident life expectancy increased from 1 to 3 years, both weekly and twice-weekly screening ICERs generally remained less than $150 000 per life-year at high levels of booster uptake and low levels of community incidence, even at 100% antiviral uptake (eFigure 1 in Supplement 1). Increasing the resident life expectancy to 5 years exhibited the same trend (eFigure 2 in Supplement 1). Additional masking scenarios and varying resident life expectancies were explored (eFigures 3-11 in Supplement 1). The cost-effectiveness of screening decreased with test sensitivity (eTable 1 in Supplement 1). Screening was less cost-effective under a more socially restricted nursing home population (eTable 2 in Supplement 1) and more cost-effective under a more socially active population (eTable 3 in Supplement 1). When only staff were screened, ICERs greatly reduced in magnitude, with only a slight increase in resident infections (eTable 4 in Supplement 1), suggesting that interventions targeting staff are highly effective, in line with findings from other studies.^17,19^

## Discussion

With the end of the COVID-19 national public health emergency^55^ and global health emergency,^56^ the world is transitioning from pandemic response to management of endemic COVID-19. However, nursing home residents are still among the most vulnerable to severe COVID-19 outcomes and experience disproportionate levels of COVID-19 mortality, even when overall case rates are low. As such, it may be valuable to continue to implement interventions in nursing homes to mitigate SARS-CoV-2 spread. Since the start of the COVID-19 pandemic in March 2020,^57^ nursing homes have sought to minimize mortality while limiting restrictive regulations that negatively affect the mental health and well-being of both residents and staff.^58,59,60^ Interventions, such as screening, vaccination, and antiviral use, are less restrictive ways to reduce resident deaths. Additionally, these strategies can substitute for one another; for example, at high levels of booster and antiviral uptake, screening may be scaled down or phased out.

### Limitations

First, we did not model the movement of residents in or out of the nursing home, which may contribute to increased transmission in the nursing home setting, particularly if residents participate in community activities where SARS-CoV-2 is more prevalent. We encourage nursing home administrators to factor in the frequency of resident turnover when considering whether to implement screening. We did not model cross-facility spread among staff who work at multiple facilities. This common practice among direct care workers may further increase the likelihood of transmission.^61^ As such, the results of this study may be conservative in the estimated transmission that would occur in a nursing home. Additionally, as we only examine resident outcomes in this cost-effectiveness analysis, those results are robust to staff turnover because the probability of transmitting SARS-CoV-2 to residents would not change over time. We did not account for changes in viral load throughout an infection, which can affect the level of rapid antigen test sensitivity depending on when a test is taken.^62,63^

In addition, we used recommended nurse staffing levels^21^ to construct the nursing home population in the model due to the heterogeneity in staffing across the country. Although nursing home staff and administrators have reported staffing shortages exacerbated by the COVID-19 pandemic,^64,65^ the results of this study show that screening can be an effective strategy in preventing SARS-CoV-2 infections among staff, which may reduce staff absences and shortages. We do not account for the potential of false-positive test results given the high specificity of rapid antigen tests^66^ but believe the trade-offs of lost staff hours due to false-positive results should be explored. Because enacting a screening strategy requires staff time and necessitates the absence of a staff member with positive test results, we encourage nursing home administrators to consider staff availability and shortages as important factors when deciding whether or not to implement screening.

As of the end of the COVID-19 public health emergency in May 2023,^55^ the CDC has stopped collecting community-level COVID-19 case counts,^67^ making it difficult for nursing homes to assess the level of SARS-CoV-2 spread in their communities using the metric we used in the model, community incidence (number of cases per 100 000 population per day). Cases are still reported by some localities, and the CDC continues to collect test result positivity, hospitalization, and death data, which may be used in place of case counts to measure community SARS-CoV-2 spread. We do not consider hospitalization costs in the present analysis, so the estimates may be conservative. However, the average national hospitalization rate for COVID-19 cases among nursing home residents in 2023 was 3.3%,^4^ suggesting that hospitalizations in this population have a limited association with overall costs. Finally, our model did not capture regional or racial differences in COVID-19 mortality rates, vaccine uptake, and antiviral use.

## Conclusions

This cost-effectiveness analysis demonstrated that, in the current Omicron era, screening is an effective strategy to reduce SARS-CoV-2 spread within nursing homes but may not be a cost-effective intervention in some situations. When community incidence is low and booster shot uptake is high, screening paired with high antiviral uptake and masking adherence may cost more economically and administratively than it is worth. However, when less of the nursing home population remains current with recommended vaccines,^68^ vaccine effectiveness continues to wane,^69^ and antiviral use remains low,^13^ screening remains an important tool in preventing severe outcomes, particularly in communities with low rates of antiviral uptake and masking adherence. As regular screening is currently at the discretion of individual nursing home facilities,^16^ we recommend that nursing homes consider implementing screening as a strategy to reduce SARS-CoV-2 spread in the absence of other interventions that would otherwise render the adoption of screening cost-ineffective.

This study provides a flexible framework for reducing COVID-19 mortality in the Omicron era in the context of unexpected waves of COVID-19 cases,^70^ rapidly mutating variants,^71^ and the recurrence of diminishing vaccine immunity.^69,72,73,74^ These findings can be used by nursing home administrators to guide planning in the context of widely varying levels of SARS-CoV-2 transmission and intervention measures across the US. Moreover, SARS-CoV-2 is the most severe respiratory virus currently in circulation,^75^ but other viruses such as influenza and respiratory syncytial virus also pose a risk to nursing home residents. The interventions modeled in this study may have a positive spillover effect in reducing the spread of other respiratory viruses.

## Notes

Supplement 1. **eMethods 1.** Calculations **eMethods 2.** Parameterization **eFigure 1.** Incremental cost of screening per resident life-year gained with antiviral treatments, assuming a 3-year life expectancy and 0.7 reduced transmission risk from staff masking **eFigure 2.** Incremental cost of screening per resident life-year gained with antiviral treatments, assuming a 5-year life expectancy and 0.7 reduced transmission risk from staff masking **eFigure 3.** Incremental cost of screening per resident life-year gained with antiviral treatments, assuming a 1-year life expectancy and 0.3 reduced transmission risk from residents, staff, and visitors masking **eFigure 4.** Incremental cost of screening per resident life-year gained with antiviral treatments, assuming a 3-year life expectancy and 0.3 reduced transmission risk from residents, staff, and visitors masking **eFigure 5.** Incremental cost of screening per resident life-year gained with antiviral treatments, assuming a 5-year life expectancy and 0.3 reduced transmission risk from residents, staff, and visitors masking **eFigure 6.** Incremental cost of screening per resident life-year gained with antiviral treatments, assuming a 1-year life expectancy and 0.7 reduced transmission risk from residents, staff, and visitors masking **eFigure 7.** Incremental cost of screening per resident life-year gained with antiviral treatments, assuming a 3-year life expectancy and 0.7 reduced transmission risk from residents, staff, and visitors masking **eFigure 8.** Incremental cost of screening per resident life-year gained with antiviral treatments, assuming a 5-year life expectancy and 0.7 reduced transmission risk from residents, staff, and visitors masking **eFigure 9.** Incremental cost of screening per resident life-year gained with antiviral treatments, assuming a 1-year life expectancy and no masking **eFigure 10.** Incremental cost of screening per resident life-year gained with antiviral treatments, assuming a 3-year life expectancy and no masking **eFigure 11.** Incremental cost of screening per resident life-year gained with antiviral treatments, assuming a 5-year life expectancy and no masking **eTable 1.** Incremental cost of screening per resident life-year gained, lowering rapid antigen test sensitivity to 0.65 **eTable 2.** Incremental cost of screening per resident life-year gained, decreasing the number of contacts residents and staff interact with in communal areas by one-third **eTable 3.** Incremental cost of screening per resident life-year gained, increasing the number of contacts residents and staff interact with in communal areas by one-third **eTable 4.** Incremental cost of screening per resident life-year gained, only staff are screened
eReferences

Supplement 2. Data Sharing Statement
