# Supplementary material for: Screening Strategies to Reduce COVID-19 Mortality in Nursing Homes
Source: JAMA Health Forum. 2024 Apr 26;5(4):e240688. doi: 10.1001/jamahealthforum.2024.0688 (PMC11065177; doi:10.1001/jamahealthforum.2024.0688)
Supplement: Supplement 1. — eMethods 1. Calculations eMethods 2. Parameterization eFigure 1. Incremental cost of screening per resident life-year gained with antiviral treatments, assuming a 3-year life expectancy and 0.7 reduced transmission risk from staff masking eFigure 2. Incremental cost of screening per resident life-year gained with antiviral treatments, assuming a 5-year life expectancy and 0.7 reduced transmission risk from staff masking eFigure 3. Incremental cost of screening per resident life-year gained with antiviral treatments, assuming a 1-year life expectancy and 0.3 reduced transmission risk from residents, staff, and visitors masking eFigure 4. Incremental cost of screening per resident life-year gained with antiviral treatments, assuming a 3-year life expectancy and 0.3 reduced transmission risk from residents, staff, and visitors masking eFigure 5. Incremental cost of screening per resident life-year gained with antiviral treatments, assuming a 5-year life expectancy and 0.3 reduced transmission risk from residents, staff, and visitors masking eFigure 6. Incremental cost of screening per resident life-year gained with antiviral treatments, assuming a 1-year life expectancy and 0.7 reduced transmission risk from residents, staff, and visitors masking eFigure 7. Incremental cost of screening per resident life-year gained with antiviral treatments, assuming a 3-year life expectancy and 0.7 reduced transmission risk from residents, staff, and visitors masking eFigure 8. Incremental cost of screening per resident life-year gained with antiviral treatments, assuming a 5-year life expectancy and 0.7 reduced transmission risk from residents, staff, and visitors masking eFigure 9. Incremental cost of screening per resident life-year gained with antiviral treatments, assuming a 1-year life expectancy and no masking eFigure 10. Incremental cost of screening per resident life-year gained with antiviral treatments, assuming a 3-year life expectancy and no masking eFigure 11. Increment [file jamahealthforum-e240688-s001.pdf]

Supplemental Online Content

Dong S, Jutkowitz E, Giardina J, Bilinski A. Screening strategies to reduce COVID-19 mortality in nursing homes. *JAMA Health Forum*. 2024;5(4):e240688. doi:10.1001/jamahealthforum.2024.0688

eMethods 1. Calculations
eMethods 2. Parameterization
eFigure 1. Incremental cost of screening per resident life-year gained with antiviral treatments, assuming a 3-year life expectancy and 0.7 reduced transmission risk from staff masking
eFigure 2. Incremental cost of screening per resident life-year gained with antiviral treatments, assuming a 5-year life expectancy and 0.7 reduced transmission risk from staff masking
eFigure 3. Incremental cost of screening per resident life-year gained with antiviral treatments, assuming a 1-year life expectancy and 0.3 reduced transmission risk from residents, staff, and visitors masking
eFigure 4. Incremental cost of screening per resident life-year gained with antiviral treatments, assuming a 3-year life expectancy and 0.3 reduced transmission risk from residents, staff, and visitors masking
eFigure 5. Incremental cost of screening per resident life-year gained with antiviral treatments, assuming a 5-year life expectancy and 0.3 reduced transmission risk from residents, staff, and visitors masking
eFigure 6. Incremental cost of screening per resident life-year gained with antiviral treatments, assuming a 1-year life expectancy and 0.7 reduced transmission risk from residents, staff, and visitors masking
eFigure 7. Incremental cost of screening per resident life-year gained with antiviral treatments, assuming a 3-year life expectancy and 0.7 reduced transmission risk from residents, staff, and visitors masking
eFigure 8. Incremental cost of screening per resident life-year gained with antiviral treatments, assuming a 5-year life expectancy and 0.7 reduced transmission risk from residents, staff, and visitors masking
eFigure 9. Incremental cost of screening per resident life-year gained with antiviral treatments, assuming a 1-year life expectancy and no masking
eFigure 10. Incremental cost of screening per resident life-year gained with antiviral treatments, assuming a 3-year life expectancy and no masking
eFigure 11. Incremental cost of screening per resident life-year gained with antiviral treatments, assuming a 5-year life expectancy and no masking
eTable 1. Incremental cost of screening per resident life-year gained, lowering rapid antigen test sensitivity to 0.65
eTable 2. Incremental cost of screening per resident life-year gained, decreasing the number of contacts residents and staff interact with in communal areas by one-third

© 2024 Dong S et al. *JAMA Health Forum.*

eTable 3. Incremental cost of screening per resident life-year gained, increasing the number of contacts residents and staff interact with in communal areas by one-third
eTable 4. Incremental cost of screening per resident life-year gained, only staff are screened
eReferences

This supplemental material has been provided by the authors to give readers additional information about their work.

© 2024 Dong S et al. *JAMA Health Forum.*

eMethods 1. Calculations

Infection probability

The probability of a susceptible agent becoming infected when interacting with an already infected agent is binomial. This binomial probability is calculated using the transmission probability of the infected agent and the immunity of the susceptible agent. The transmission probability of the infected agent is determined by the attack rate of the virus multiplied by a reduction factor conferred by masking (if masks are used). In "SARS-CoV-2 transmission parameters" in eMethods 2, we find the 8-hour shift transmission probability using the attack rate of Omicron. The immunity of the susceptible agent is conferred by the agent's vaccination or previous infection status (explained in "Vaccine and infection-induced immunity against infection" in eMethods 2). This immunity protection reduces an agent's susceptibility to infection, so the binomial probability of a susceptible agent becoming infected during each 8-hour shift is as follows:

$$shift\ transmission\ probability * (1 - masking\ efficacy) * (1 - vaccine\ or\ previous\ infection\ efficacy)$$

where $shift\ transmission\ probability$ is the 8-hour transmission probability of the infected agent, $(1 - masking\ efficacy)$ is the reduction in the transmission risk of an infected agent wearing a mask (and if the infected agent is not wearing a mask, the reduction in transmission risk is 0), and $(1 - vaccine\ or\ previous\ infection\ efficacy)$ is the reduction in the risk of infecting a susceptible agent due to the immunity offered by the susceptible agent's vaccination or previous infection status.

Incremental cost-effectiveness ratio

The incremental cost of screening per resident life-year gained is obtained by calculating:

$$\frac{difference\ in\ cost\ of\ screening\ compared\ to\ less\ frequent\ screening\ strategy}{difference\ in\ number\ of\ resident\ deaths\ compared\ to\ less\ frequent\ screening\ strategy}.$$

For example, the incremental cost of screening per resident life-year gained for weekly screening is:

$$\frac{cost\ of\ weekly\ screening - cost\ of\ no\ screening}{number\ of\ resident\ deaths\ with\ no\ screening - number\ of\ resident\ deaths\ with\ weekly\ screening}.$$

The cost of each rapid antigen test is assumed to be $5, based on the bulk rate of rapid antigen tests for schools[1], wholesale pricing for healthcare facilities[2,3,4,5], and estimates from other analyses[6]. We estimate that each test takes around 12 minutes to administer to residents[7], and nursing assistants who administer resident tests get paid $15.43 per hour[8]:

$$cost\ of\ screening\ = (total\ test\ count) * (\$5) + (resident\ test\ count) * (12\ minutes) * \left(\frac{1\ hour}{60\ minutes}\right) * \left(\frac{\$15.43}{1\ hour}\right).$$

The number of resident deaths is calculated by multiplying the number of resident infections with the case-fatality ratio (CFR); the calculation for the CFR is detailed in "Case-fatality ratio (CFR)" in eMethods 2.

© 2024 Dong S et al. *JAMA Health Forum.*

eMethods 2. Parameterization

Vaccine and infection-induced immunity against infection

Due to how rapidly vaccine-induced immunity wanes over time and the added complexity of infection-induced immunity, we classify each agent in the nursing home as either 1) vaccinated with the two-dose primary series or previously infected, or 2) boosted with any of the booster doses currently offered. As such, we assume that those who have been vaccinated with two doses or have been previously infected share the same level of protection against infection, and those who have been boosted with any of the booster doses share the same level of protection.

Pooling estimates of vaccine efficacy and immunity offered by previous infection against Omicron, we estimate that a two-dose vaccine series or previous infection is roughly 40% effective against infection, and any additional booster dose is about 70% effective[9].

Vaccination rates

A KFF analysis of nursing home data in September 2022 found that 74% of residents and 51% of staff had received at least one additional monovalent booster shot[10]. For the bivalent booster dose as of December 2022, uptake among residents and staff was approximately 48% and 22% respectively[11].

We also estimate the proportion of nursing home visitors vaccinated against SARS-CoV-2. We assume the general U.S. population is representative of visitors to the nursing home. According to the NYTimes COVID-19 dashboard[12], 34% of the general U.S. population aged 18-64 had received a monovalent booster dose as of October 2022. Seven percent of eligible adults as of December 2022 had received the bivalent booster dose[13].

We use the proportions of monovalent and bivalent booster shot uptake in these populations to denote low and high booster uptake respectively: low booster uptake is 48% in residents, 22% in staff, and 7% in visitors; high booster uptake is 74% in residents, 51% in staff, and 34% in visitors.

SARS-CoV-2 transmission parameters

There are multiple stages of SARS-CoV-2 infection that we parametrize in the model: the latent period, incubation period, and length of infection. The latent period of infection is the length of time between exposure to the virus and the start of infectiousness. For Omicron, we estimate that the latent period follows a gamma distribution where α = 4.45 and β = 1.42[14]. The incubation period is the length of time between exposure to the virus and the appearance of symptoms (if symptomatic). For Omicron we estimate that the incubation period follows a gamma distribution where α = 8.38 and β = 2.20[14]. We assume the length of infectiousness for an Omicron infection is 5 days on average, in accordance with CDC guidelines that advise isolating for 5 days upon notification of infection[15].

The daily transmission probability (the probability that the contact of an infected agent will become infected), 0.18, is calculated using the attack rate of unvaccinated persons (63.9%) with sequence-confirmed Omicron infections between November 2021 and February 2022[16]. We use the equation:

$$1-(1-p)^{5\ days}=0.639$$

for p = daily transmission probability and a 5-day infectious period. Solving for p, we get a baseline daily transmission probability of about 0.18. Because we run our model in 8-hour shifts, we derive the shift transmission probability with the equation:

$$1-(1-r)^{3}=0.18$$

for r = shift transmission probability and three 8-hour shifts per day. This gives us a baseline shift transmission probability of about 0.064.

© 2024 Dong S et al. *JAMA Health Forum.*

Testing parameters

We estimate that the proportion of asymptomatic infections in the nursing home is around 50%[17,18]. For individuals who are asymptomatic, the nursing home is not aware they are infectious unless and until they screen positive. For those who are symptomatic, the nursing home becomes aware they are infectious when symptoms appear or if/when they screen positive, whichever comes first. Rapid antigen test sensitivity, the likelihood that the test result of an infected individual will be positive, is estimated to be 84%[19,20]. We assume that not everyone in the nursing home is tested when screening is in place for any reason (e.g. refusal to test or inability to test) and parametrize the proportion of tested individuals in the nursing home to 90%.

Case-fatality ratio (CFR)

Using CMS data on COVID-19 in U.S. nursing homes[11], we calculate the CFR in nursing home residents, which is the proportion of residents diagnosed with COVID-19 who die from the virus. We assume there is a two-week lag between infection from SARS-CoV-2 and death from SARS-CoV-2. The CFR calculated directly from the data (number of COVID-19 deaths ÷ number of COVID-19 infections) is confounded with antiviral use, as many nursing homes have started to use antiviral treatments to prevent deaths. As such, we hope to calculate the "untreated" CFR. We take the average proportion of nursing home residents across the U.S. that received antivirals between January 2023 and April 2023, which we call the "treated proportion", and use the following formula to calculate the untreated CFR:

$$CFR = (untreated\ proportion) * (untreated\ CFR) + (treated\ proportion) * (untreated\ CFR) * (1 - antiviral\ effectiveness\ against\ death).$$

The CFR from the data is around 1.8%[11]. The treated proportion is about 32%[11]. Although we use the proportion of residents treated with both nirmatrelvir/ritonavir (22%) and molnupiravir (10%) to make up the treated proportion[11], we attribute the treated proportion's protection to nirmatrelvir/ritonavir's 71% effectiveness against death[21]:

$$0.018 = 0.68 * (untreated\ CFR) + 0.32 * (untreated\ CFR) * (1 - 0.71)$$

which gives us an untreated CFR of around 0.023.

© 2024 Dong S et al. *JAMA Health Forum.*

eFigure 1. Incremental cost of screening per resident life-year gained with antiviral treatments, assuming a 3-year life expectancy and 0.7 reduced transmission risk from staff masking

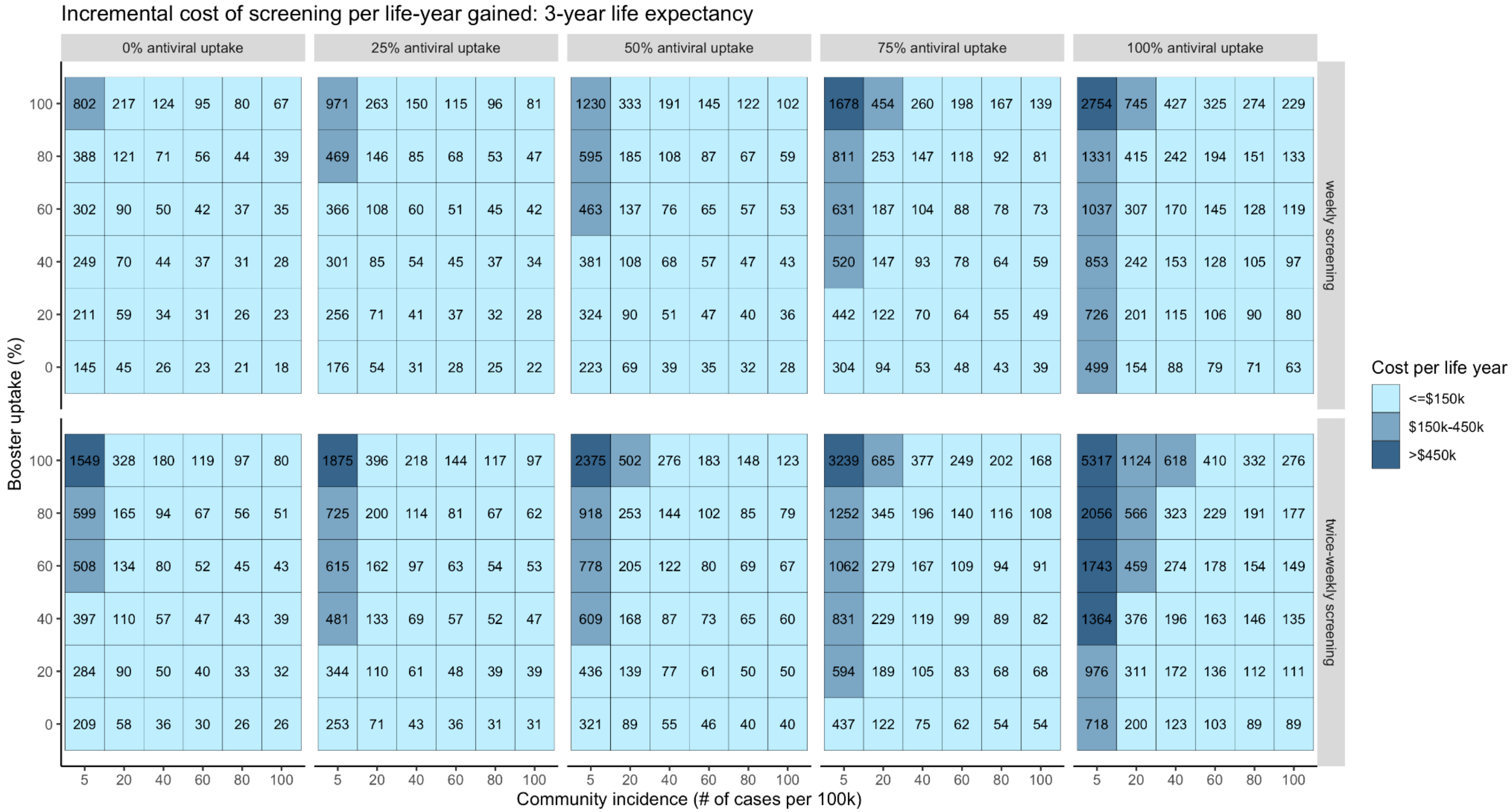

Costs are denoted in thousands of dollars, and are rounded to the nearest thousand. At 0% booster uptake, the proportion of boosted residents, staff, and visitors are all 0%. At all other levels of booster uptake, the proportion of boosted residents is the percentage on the y-axis, the proportion of boosted staff is half that of the boosted residents, and the proportion of boosted visitors is a quarter that of boosted residents (e.g. at 20% booster uptake, the proportion of boosted residents, staff, and visitors is 20%, 10%, and 5% respectively).

© 2024 Dong S et al. *JAMA Health Forum.*

eFigure 2. Incremental cost of screening per resident life-year gained with antiviral treatments, assuming a 5-year life expectancy and 0.7 reduced transmission risk from staff masking

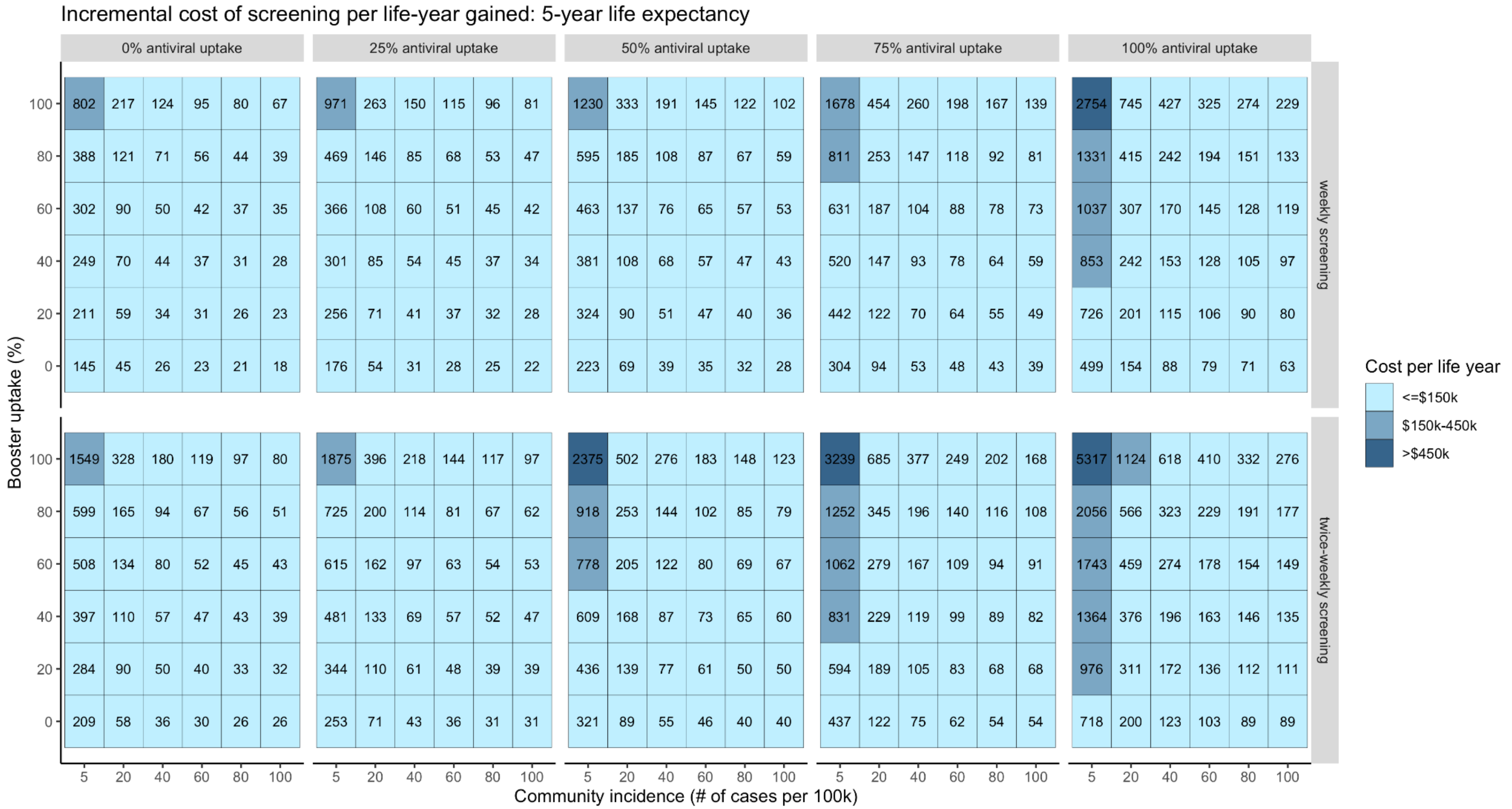

Costs are denoted in thousands of dollars, and are rounded to the nearest thousand. At 0% booster uptake, the proportion of boosted residents, staff, and visitors are all 0%. At all other levels of booster uptake, the proportion of boosted residents is the percentage on the y-axis, the proportion of boosted staff is half that of the boosted residents, and the proportion of boosted visitors is a quarter that of boosted residents (e.g. at 20% booster uptake, the proportion of boosted residents, staff, and visitors is 20%, 10%, and 5% respectively).

© 2024 Dong S et al. *JAMA Health Forum.*

eFigure 3. Incremental cost of screening per resident life-year gained with antiviral treatments, assuming a 1-year life expectancy and 0.3 reduced transmission risk from residents, staff, and visitors masking

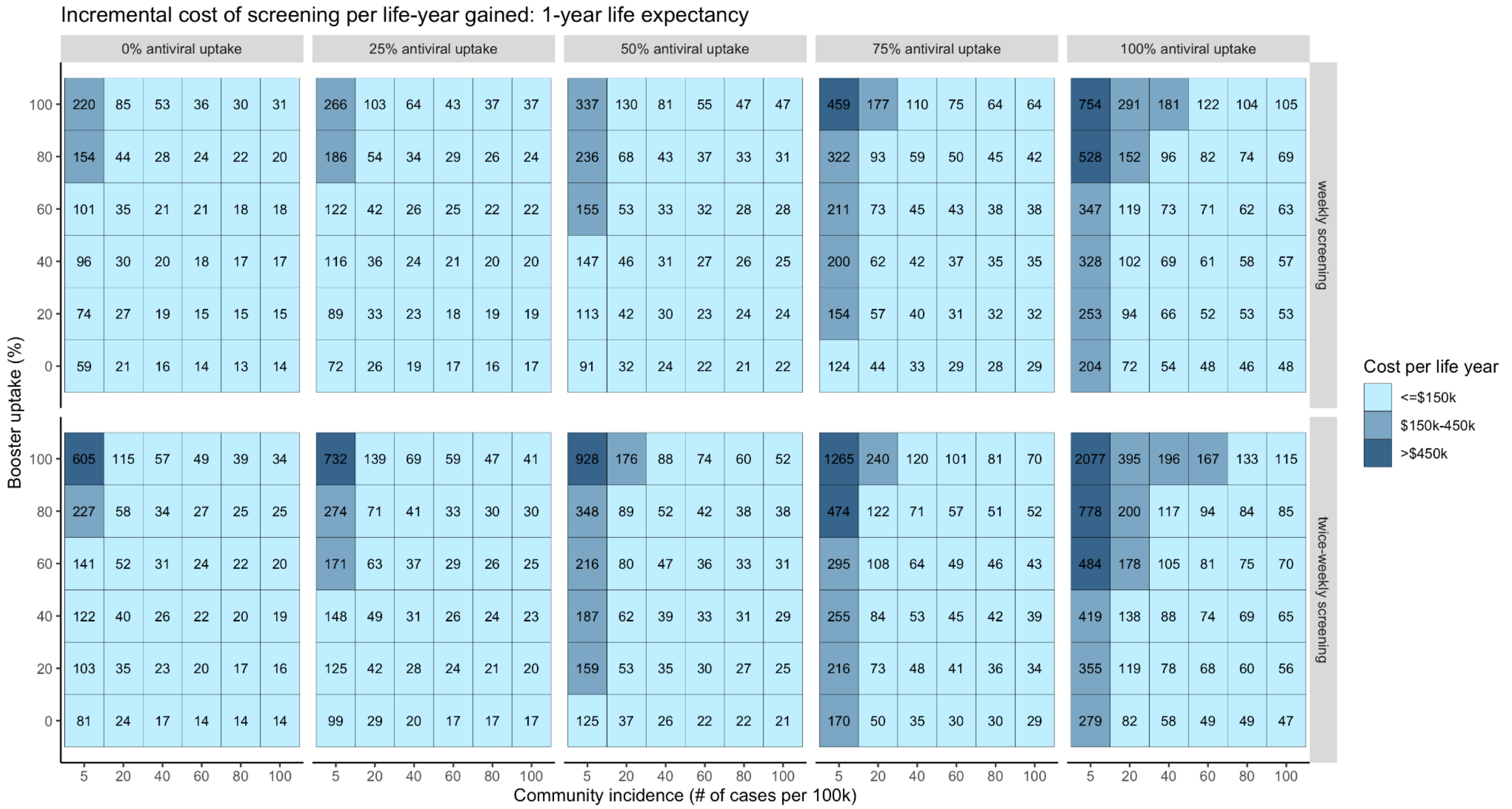

When masks are worn but best masking practices are not adhered to (e.g. infrequent masking or wearing masks improperly), we assume that infected agents' transmission risk is lowered by 0.3. Costs are denoted in thousands of dollars, and are rounded to the nearest thousand. At 0% booster uptake, the proportion of boosted residents, staff, and visitors are all 0%. At all other levels of booster uptake, the proportion of boosted residents is the percentage on the y-axis, the proportion of boosted staff is half that of the boosted residents, and the proportion of boosted visitors is a quarter that of boosted residents (e.g. at 20% booster uptake, the proportion of boosted residents, staff, and visitors is 20%, 10%, and 5% respectively).

© 2024 Dong S et al. *JAMA Health Forum.*

eFigure 4. Incremental cost of screening per resident life-year gained with antiviral treatments, assuming a 3-year life expectancy and 0.3 reduced transmission risk from residents, staff, and visitors masking

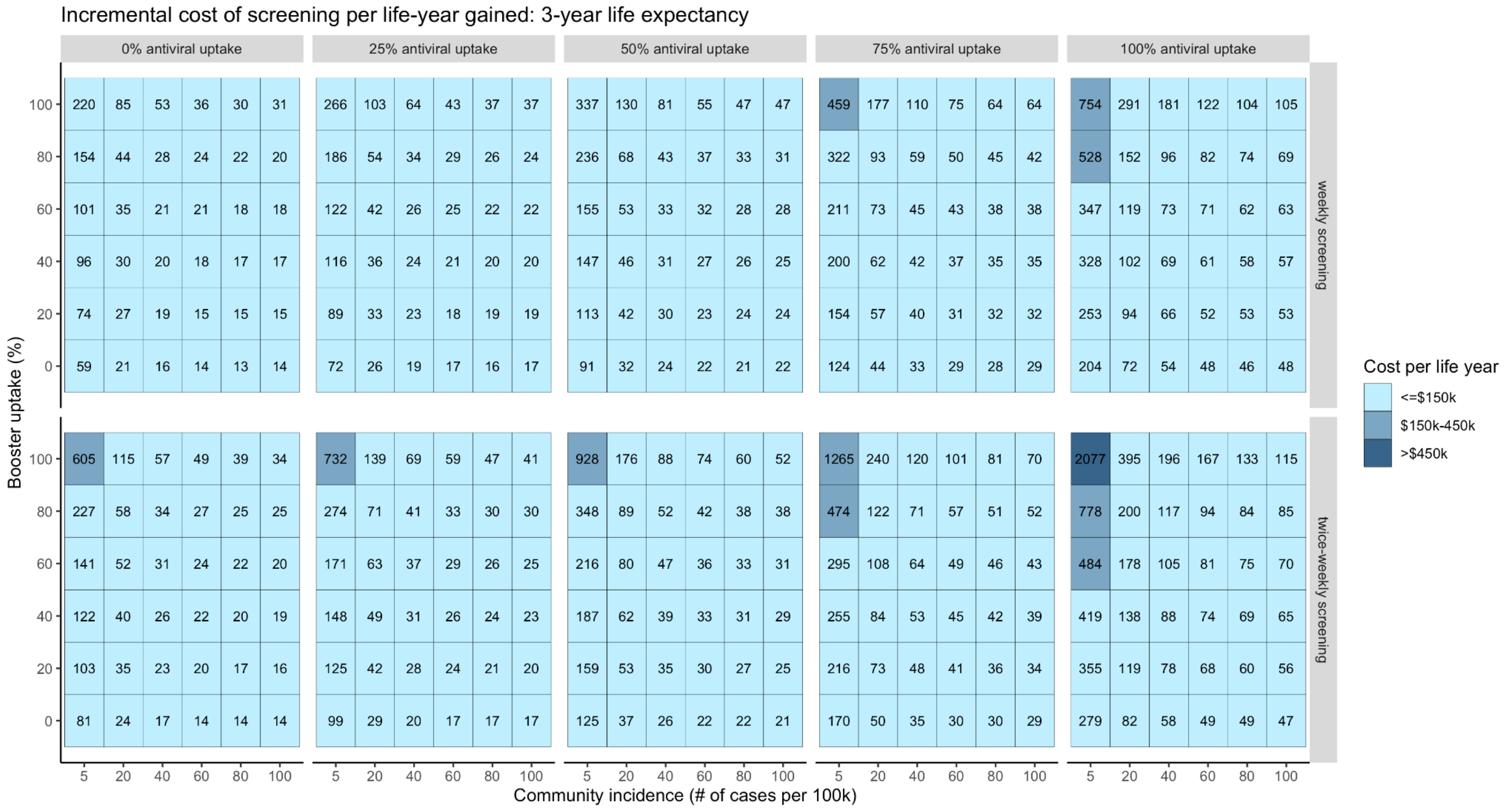

When masks are worn but best masking practices are not adhered to (e.g. infrequent masking or wearing masks improperly), we assume that infected agents' transmission risk is lowered by 0.3. Costs are denoted in thousands of dollars, and are rounded to the nearest thousand. At 0% booster uptake, the proportion of boosted residents, staff, and visitors are all 0%. At all other levels of booster uptake, the proportion of boosted residents is the percentage on the y-axis, the proportion of boosted staff is half that of the boosted residents, and the proportion of boosted visitors is a quarter that of boosted residents (e.g. at 20% booster uptake, the proportion of boosted residents, staff, and visitors is 20%, 10%, and 5% respectively).

© 2024 Dong S et al. *JAMA Health Forum.*

eFigure 5. Incremental cost of screening per resident life-year gained with antiviral treatments, assuming a 5-year life expectancy and 0.3 reduced transmission risk from residents, staff, and visitors masking

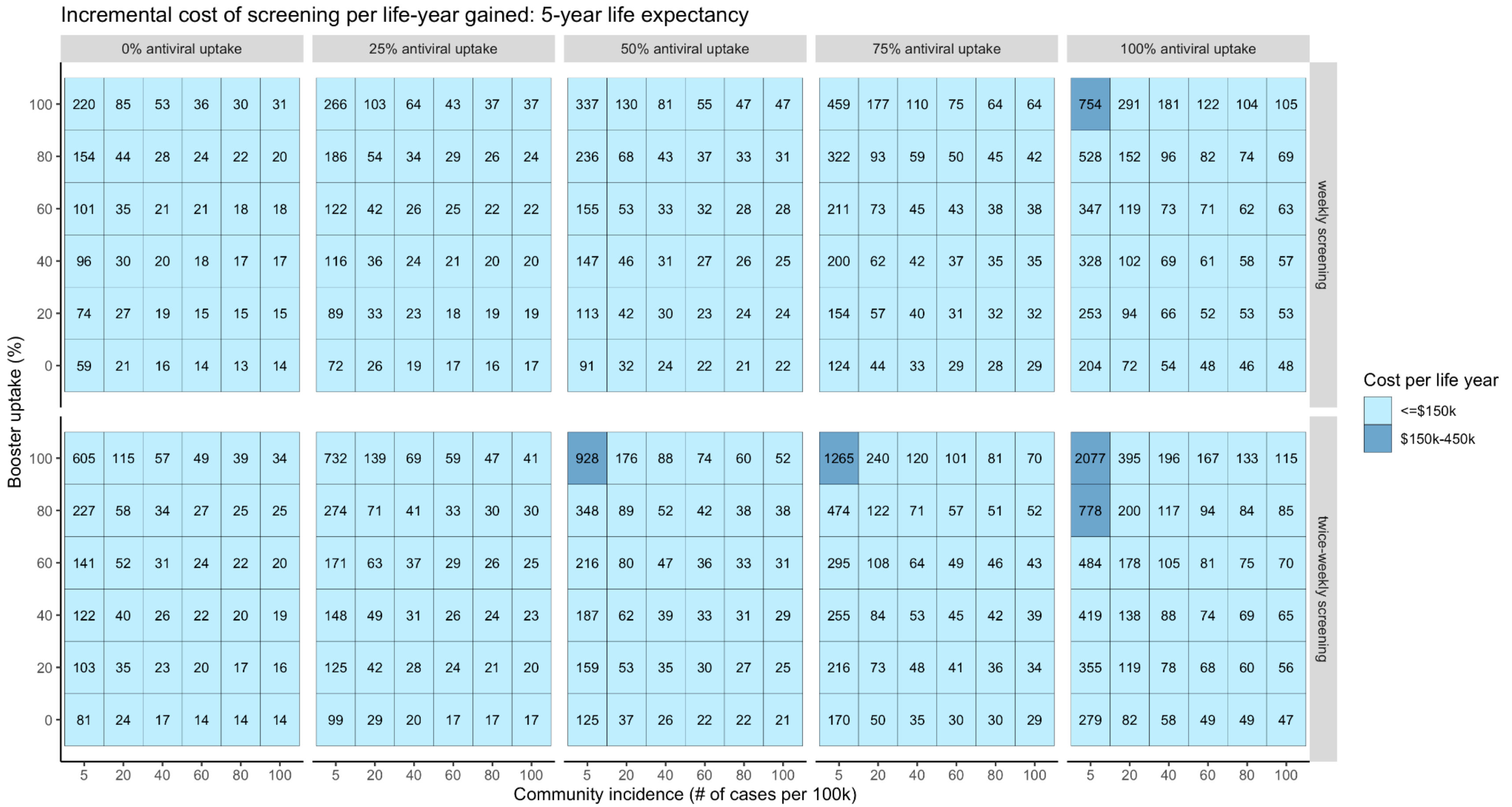

When masks are worn but best masking practices are not adhered to (e.g. infrequent masking or wearing masks improperly), we assume that infected agents' transmission risk is lowered by 0.3. Costs are denoted in thousands of dollars, and are rounded to the nearest thousand. At 0% booster uptake, the proportion of boosted residents, staff, and visitors are all 0%. At all other levels of booster uptake, the proportion of boosted residents is the percentage on the y-axis, the proportion of boosted staff is half that of the boosted residents, and the proportion of boosted visitors is a quarter that of boosted residents (e.g. at 20% booster uptake, the proportion of boosted residents, staff, and visitors is 20%, 10%, and 5% respectively).

© 2024 Dong S et al. *JAMA Health Forum.*

eFigure 6. Incremental cost of screening per resident life-year gained with antiviral treatments, assuming a 1-year life expectancy and 0.7 reduced transmission risk from residents, staff, and visitors masking

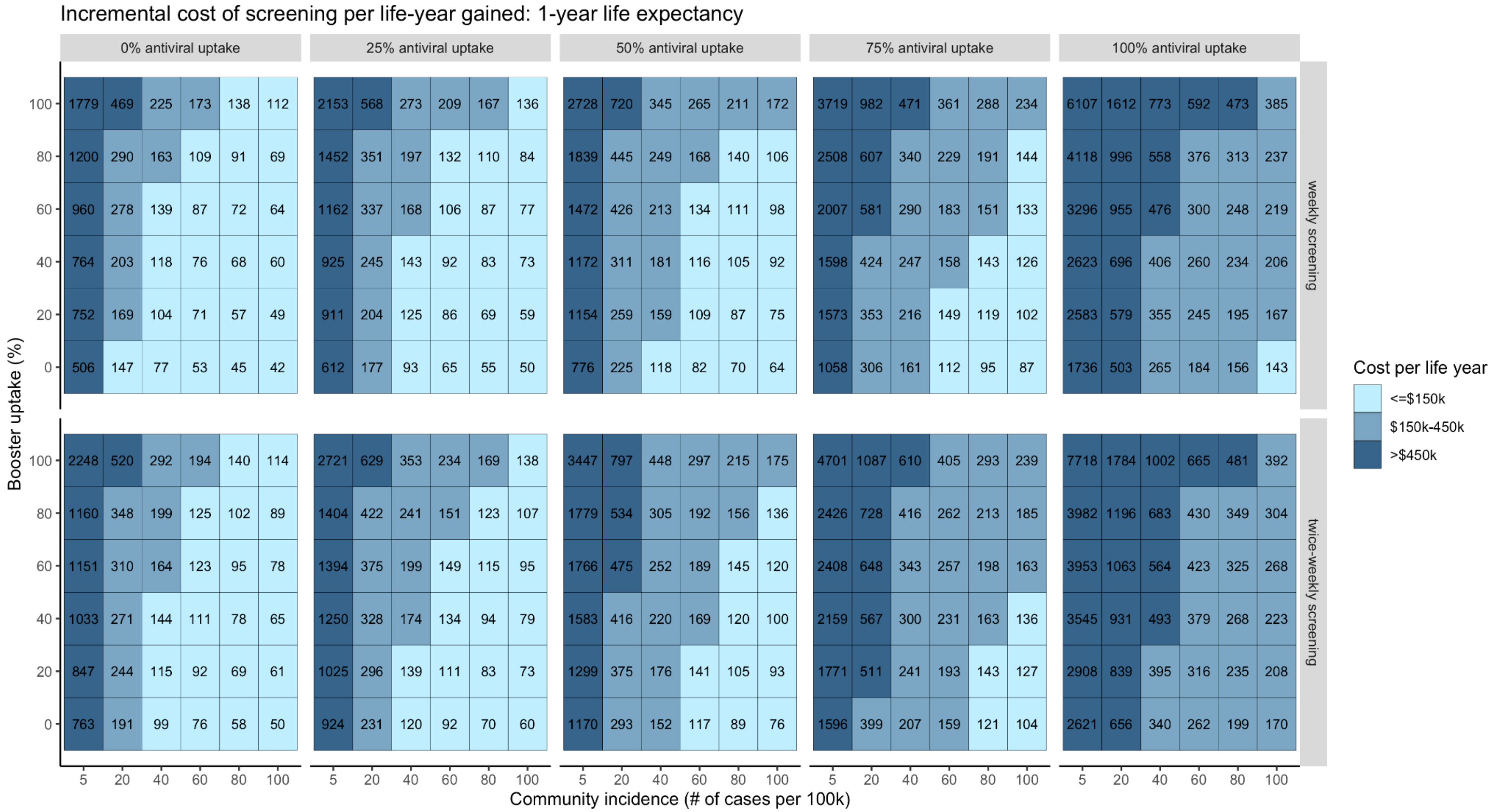

Costs are denoted in thousands of dollars, and are rounded to the nearest thousand. At 0% booster uptake, the proportion of boosted residents, staff, and visitors are all 0%. At all other levels of booster uptake, the proportion of boosted residents is the percentage on the y-axis, the proportion of boosted staff is half that of the boosted residents, and the proportion of boosted visitors is a quarter that of boosted residents (e.g. at 20% booster uptake, the proportion of boosted residents, staff, and visitors is 20%, 10%, and 5% respectively).

© 2024 Dong S et al. *JAMA Health Forum.*

eFigure 7. Incremental cost of screening per resident life-year gained with antiviral treatments, assuming a 3-year life expectancy and 0.7 reduced transmission risk from residents, staff, and visitors masking

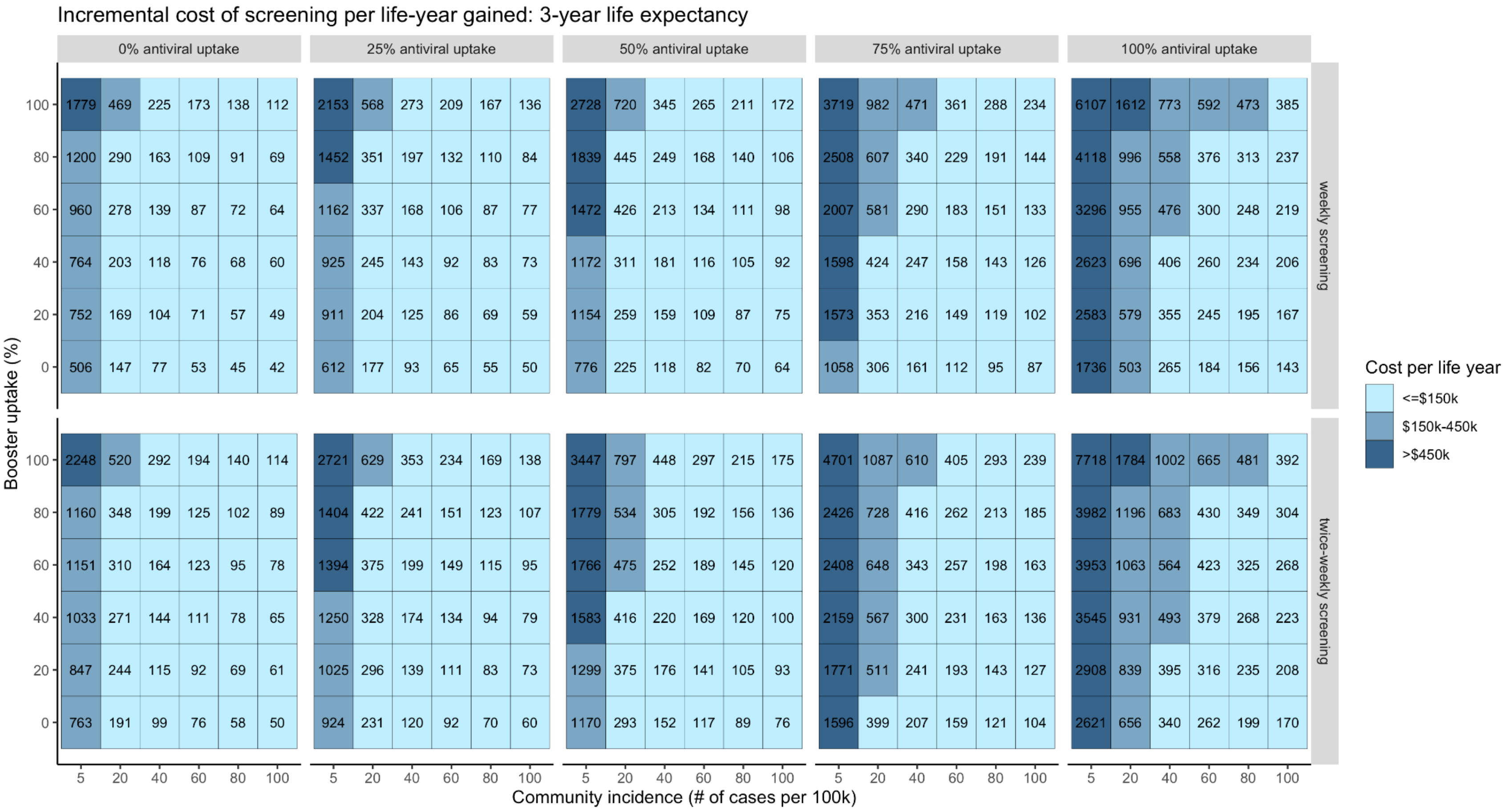

Costs are denoted in thousands of dollars, and are rounded to the nearest thousand. At 0% booster uptake, the proportion of boosted residents, staff, and visitors are all 0%. At all other levels of booster uptake, the proportion of boosted residents is the percentage on the y-axis, the proportion of boosted staff is half that of the boosted residents, and the proportion of boosted visitors is a quarter that of boosted residents (e.g. at 20% booster uptake, the proportion of boosted residents, staff, and visitors is 20%, 10%, and 5% respectively).

© 2024 Dong S et al. *JAMA Health Forum.*

eFigure 8. Incremental cost of screening per resident life-year gained with antiviral treatments, assuming a 5-year life expectancy and 0.7 reduced transmission risk from residents, staff, and visitors masking

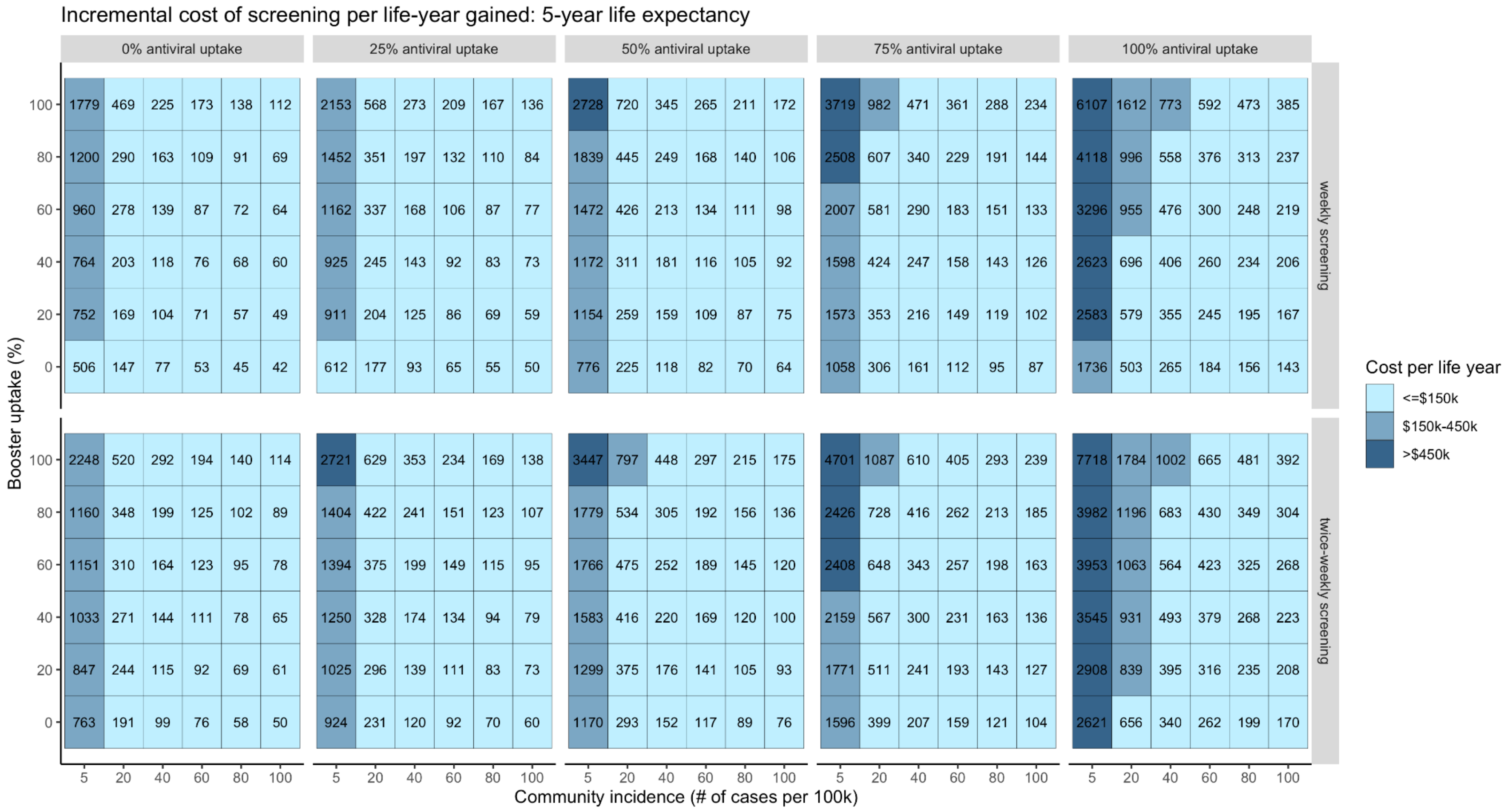

Costs are denoted in thousands of dollars, and are rounded to the nearest thousand. At 0% booster uptake, the proportion of boosted residents, staff, and visitors are all 0%. At all other levels of booster uptake, the proportion of boosted residents is the percentage on the y-axis, the proportion of boosted staff is half that of the boosted residents, and the proportion of boosted visitors is a quarter that of boosted residents (e.g. at 20% booster uptake, the proportion of boosted residents, staff, and visitors is 20%, 10%, and 5% respectively).

© 2024 Dong S et al. *JAMA Health Forum.*

eFigure 9. Incremental cost of screening per resident life-year gained with antiviral treatments, assuming a 1-year life expectancy and no masking

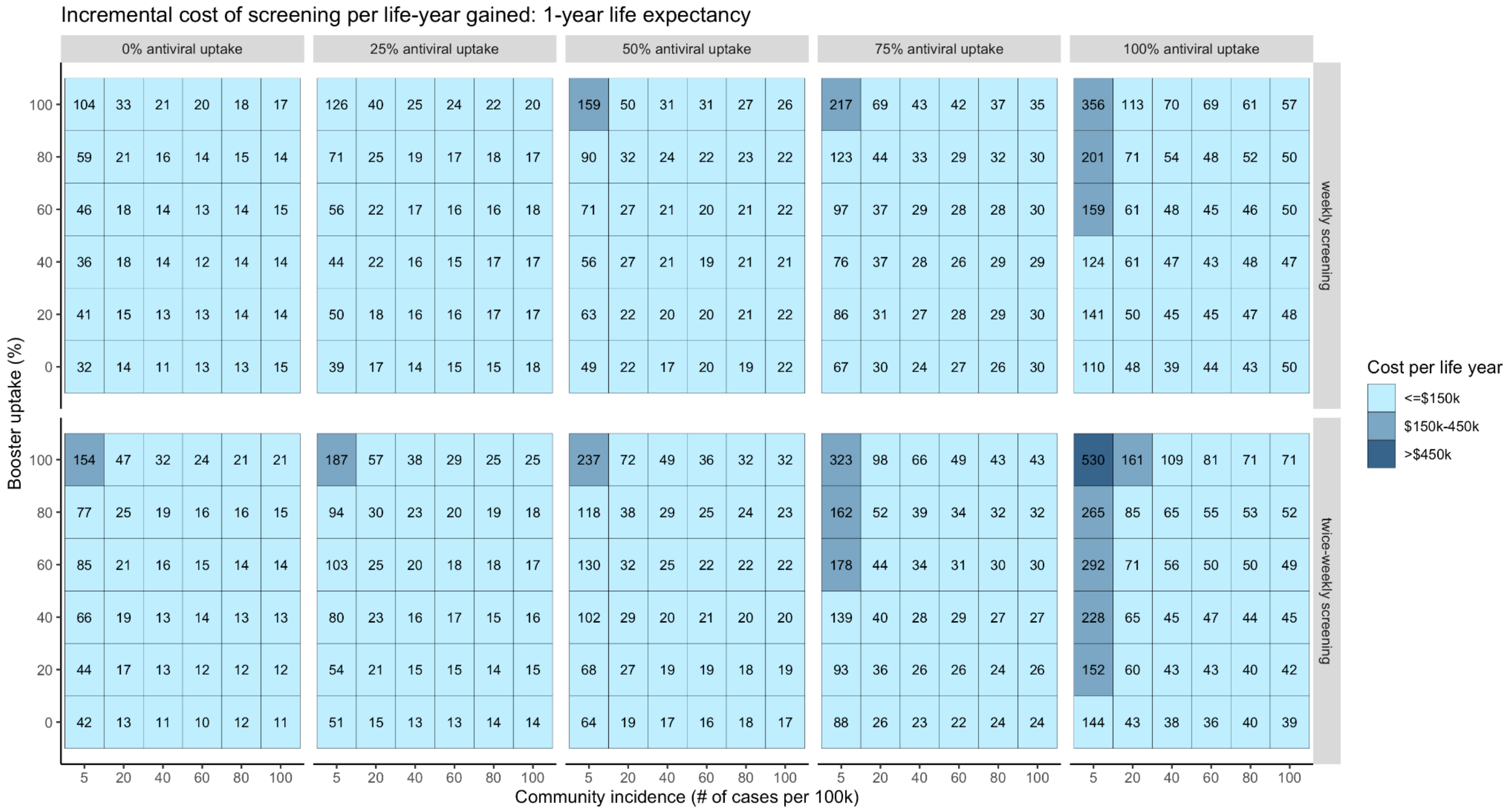

Costs are denoted in thousands of dollars, and are rounded to the nearest thousand. At 0% booster uptake, the proportion of boosted residents, staff, and visitors are all 0%. At all other levels of booster uptake, the proportion of boosted residents is the percentage on the y-axis, the proportion of boosted staff is half that of the boosted residents, and the proportion of boosted visitors is a quarter that of boosted residents (e.g. at 20% booster uptake, the proportion of boosted residents, staff, and visitors is 20%, 10%, and 5% respectively).

© 2024 Dong S et al. *JAMA Health Forum.*

eFigure 10. Incremental cost of screening per resident life-year gained with antiviral treatments, assuming a 3-year life expectancy and no masking

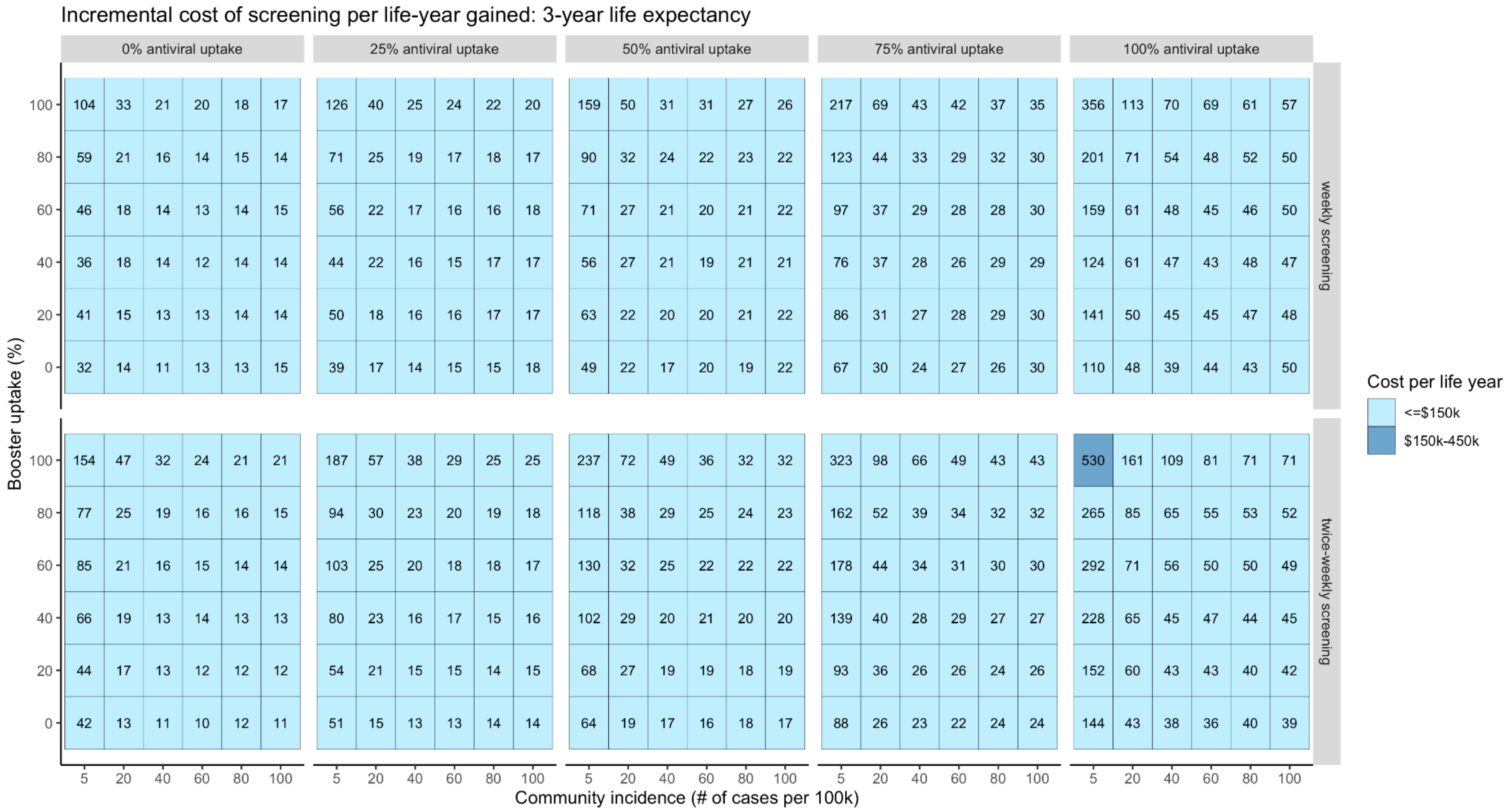

Costs are denoted in thousands of dollars, and are rounded to the nearest thousand. At 0% booster uptake, the proportion of boosted residents, staff, and visitors are all 0%. At all other levels of booster uptake, the proportion of boosted residents is the percentage on the y-axis, the proportion of boosted staff is half that of the boosted residents, and the proportion of boosted visitors is a quarter that of boosted residents (e.g. at 20% booster uptake, the proportion of boosted residents, staff, and visitors is 20%, 10%, and 5% respectively).

© 2024 Dong S et al. *JAMA Health Forum.*

eFigure 11. Incremental cost of screening per resident life-year gained with antiviral treatments, assuming a 5-year life expectancy and no masking

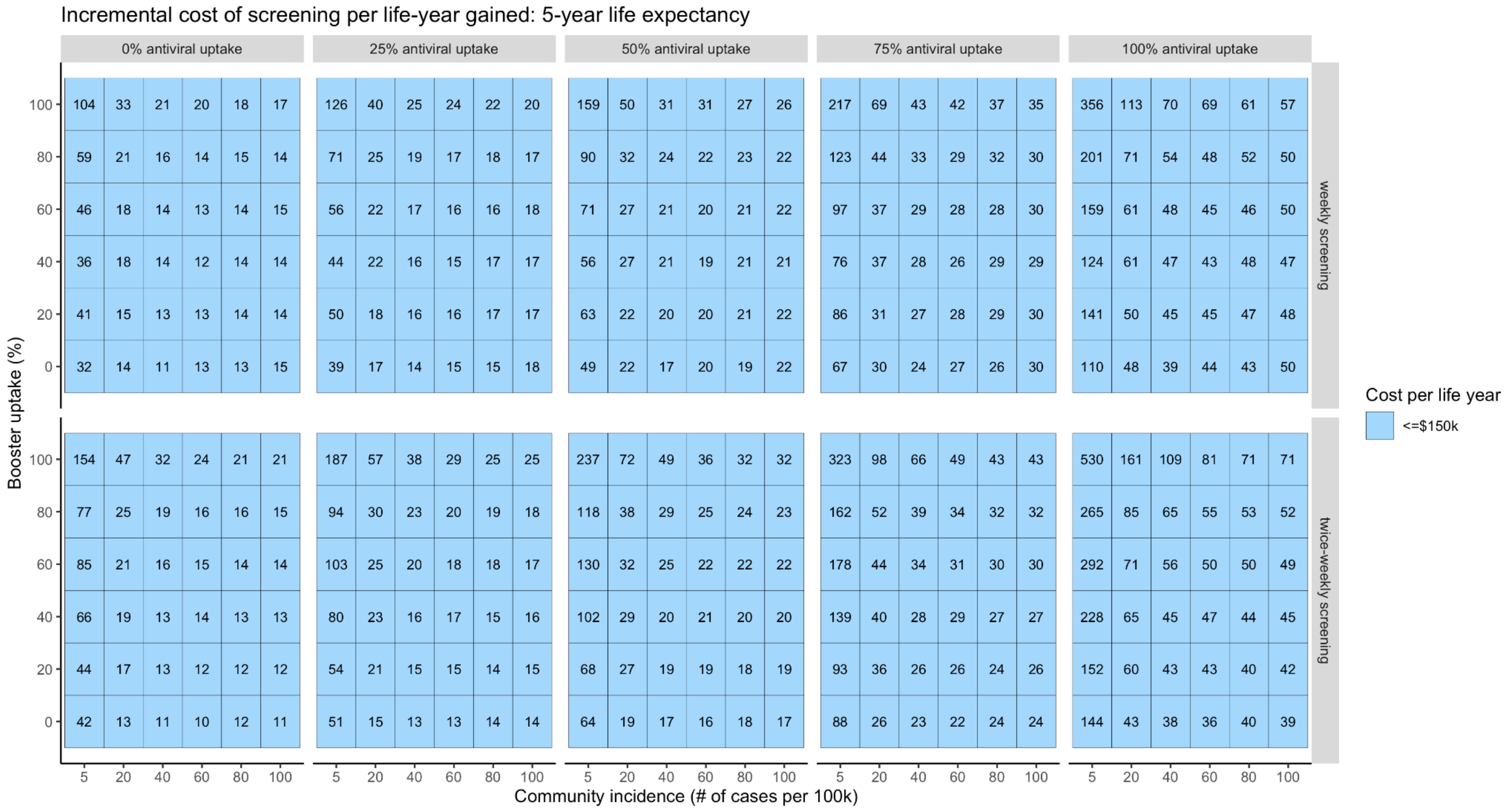

Costs are denoted in thousands of dollars, and are rounded to the nearest thousand. At 0% booster uptake, the proportion of boosted residents, staff, and visitors are all 0%. At all other levels of booster uptake, the proportion of boosted residents is the percentage on the y-axis, the proportion of boosted staff is half that of the boosted residents, and the proportion of boosted visitors is a quarter that of boosted residents (e.g. at 20% booster uptake, the proportion of boosted residents, staff, and visitors is 20%, 10%, and 5% respectively).

© 2024 Dong S et al. *JAMA Health Forum.*

eTable 1. Incremental cost of screening per resident life-year gained, lowering rapid antigen test sensitivity to 0.65

	Low booster uptake					High booster uptake				
Strategy	Cost	Incremental cost	Resident deaths	Incremental reduction in deaths	Incremental cost per resident life-year gained	Cost	Incremental cost	Resident deaths	Incremental reduction in deaths	Incremental cost per resident life-year gained
Community incidence: 5 per 100,000						Community incidence: 5 per 100,000				
No screening	$0	-	0.027	-	-	$0	-	0.020	-	-
Weekly screening	$4000	$4000	0.019	0.0077	$533,000	$4000	$4000	0.014	0.0053	$776,000
Twice-weekly screening	$8000	$4000	0.012	0.0065	$631,000	$8000	$4000	0.0096	0.0047	$878,000
Community incidence: 50 per 100,000						Community incidence: 50 per 100,000				
No screening	$0	-	0.21	-	-	$0	-	0.16	-	-
Weekly screening	$4000	$4000	0.15	0.054	$76,000	$4000	$4000	0.12	0.041	$100,000
Twice-weekly screening	$8000	$4000	0.11	0.048	$85,000	$8000	$4000	0.083	0.038	$108,000
Community incidence: 100 per 100,000						Community incidence: 100 per 100,000				
No screening	$0	-	0.32	-	-	$0	-	0.26	-	-
Weekly screening	$4000	$4000	0.25	0.075	$54,000	$4000	$4000	0.20	0.063	$65,000
Twice-weekly screening	$8000	$4000	0.18	0.069	$59,000	$8000	$4000	0.14	0.054	$76,000

Costs are rounded to the nearest thousand, and deaths are rounded to two significant digits. The incremental cost-effectiveness ratios presented in this table were obtained with calculations using exact values, not the rounded values presented in the table. Low, moderate, and high community incidence (number of cases per 100,000 population per day) are denoted as 5, 50, and 100 per 100,000 respectively.

© 2024 Dong S et al. *JAMA Health Forum.*

eTable 2. Incremental cost of screening per resident life-year gained, decreasing the number of contacts residents and staff interact with in communal areas by one-third

	Low booster uptake					High booster uptake				
Strategy	Cost	Incremental cost	Resident deaths	Incremental reduction in deaths	Incremental cost per resident life-year gained	Cost	Incremental cost	Resident deaths	Incremental reduction in deaths	Incremental cost per resident life-year gained
Community incidence: 5 per 100,000						Community incidence: 5 per 100,000				
No screening	$0	-	0.025	-	-	$0	-	0.018	-	-
Weekly screening	$4000	$4000	0.014	0.011	$386,000	$4000	$4000	0.012	0.0058	$704,000
Twice-weekly screening	$8000	$4000	0.0087	0.0055	$750,000	$8000	$4000	0.0068	0.0052	$796,000
Community incidence: 50 per 100,000						Community incidence: 50 per 100,000				
No screening	$0	-	0.18	-	-	$0	-	0.14	-	-
Weekly screening	$4000	$4000	0.13	0.058	$70,000	$4000	$4000	0.095	0.048	$86,000
Twice-weekly screening	$8000	$4000	0.077	0.049	$83,000	$8000	$4000	0.063	0.032	$126,000
Community incidence: 100 per 100,000						Community incidence: 100 per 100,000				
No screening	$0	-	0.30	-	-	$0	-	0.23	-	-
Weekly screening	$4000	$4000	0.21	0.092	$44,000	$4000	$4000	0.17	0.067	$61,000
Twice-weekly screening	$8000	$4000	0.14	0.071	$57,000	$8000	$4000	0.12	0.052	$78,000

Costs are rounded to the nearest thousand, and deaths are rounded to two significant digits. The incremental cost-effectiveness ratios presented in this table were obtained with calculations using exact values, not the rounded values presented in the table. Low, moderate, and high community incidence (number of cases per 100,000 population per day) are denoted as 5, 50, and 100 per 100,000 respectively.

© 2024 Dong S et al. *JAMA Health Forum.*

eTable 3. Incremental cost of screening per resident life-year gained, increasing the number of contacts residents and staff interact with in communal areas by one-third

	Low booster uptake					High booster uptake				
Strategy	Cost	Incremental cost	Resident deaths	Incremental reduction in deaths	Incremental cost per resident life-year gained	Cost	Incremental cost	Resident deaths	Incremental reduction in deaths	Incremental cost per resident life-year gained
Community incidence: 5 per 100,000						Community incidence: 5 per 100,000				
No screening	$0	-	0.032	-	-	$0	-	0.022	-	-
Weekly screening	$4000	$4000	0.019	0.012	$334,000	$4000	$4000	0.014	0.0084	$488,000
Twice-weekly screening	$8000	$4000	0.011	0.0087	$474,000	$8000	$4000	0.0081	0.0059	$693,000
Community incidence: 50 per 100,000						Community incidence: 50 per 100,000				
No screening	$0	-	0.23	-	-	$0	-	0.18	-	-
Weekly screening	$4000	$4000	0.15	0.083	$49,000	$4000	$4000	0.12	0.067	$61,000
Twice-weekly screening	$8000	$4000	0.090	0.058	$70,000	$8000	$4000	0.070	0.045	$91,000
Community incidence: 100 per 100,000						Community incidence: 100 per 100,000				
No screening	$0	-	0.36	-	-	$0	-	0.29	-	-
Weekly screening	$4000	$4000	0.24	0.12	$33,000	$4000	$4000	0.20	0.093	$44,000
Twice-weekly screening	$8000	$4000	0.15	0.087	$47,000	$8000	$4000	0.13	0.071	$57,000

Costs are rounded to the nearest thousand, and deaths are rounded to two significant digits. The incremental cost-effectiveness ratios presented in this table were obtained with calculations using exact values, not the rounded values presented in the table. Low, moderate, and high community incidence (number of cases per 100,000 population per day) are denoted as 5, 50, and 100 per 100,000 respectively.

© 2024 Dong S et al. *JAMA Health Forum.*

eTable 4. Incremental cost of screening per resident life-year gained, only staff are screened

	Low booster uptake					High booster uptake				
Strategy	Cost	Incremental cost	Resident deaths	Incremental reduction in deaths	Incremental cost per resident life-year gained	Cost	Incremental cost	Resident deaths	Incremental reduction in deaths	Incremental cost per resident life-year gained
Community incidence: 5 per 100,000						Community incidence: 5 per 100,000				
No screening	$0	-	0.025	-	-	$0	-	0.020	-	-
Weekly screening	$1500	$1500	0.018	0.0070	$213,500	$1500	$1500	0.014	0.0061	$243,500
Twice-weekly screening	$3000	$1500	0.012	0.0064	$232,500	$3000	$1500	0.0083	0.0053	$283,500
Community incidence: 50 per 100,000						Community incidence: 50 per 100,000				
No screening	$0	-	0.21	-	-	$0	-	0.16	-	-
Weekly screening	$1500	$1500	0.15	0.058	$25,500	$1500	$1500	0.12	0.043	$34,500
Twice-weekly screening	$3000	$1500	0.098	0.050	$29,500	$3000	$1500	0.077	0.039	$38,000
Community incidence: 100 per 100,000						Community incidence: 100 per 100,000				
No screening	$0	-	0.32	-	-	$0	-	0.27	-	-
Weekly screening	$1500	$1500	0.24	0.079	$18,500	$1500	$1500	0.20	0.069	$21,500
Twice-weekly screening	$3000	$1500	0.17	0.074	$19,500	$3000	$1500	0.14	0.059	$24,500

Costs are rounded to the nearest multiple of 500, and deaths are rounded to two significant digits. The incremental cost-effectiveness ratios presented in this table were obtained with calculations using exact values, not the rounded values presented in the table. Low, moderate, and high community incidence (number of cases per 100,000 population per day) are denoted as 5, 50, and 100 per 100,000 respectively.

© 2024 Dong S et al. *JAMA Health Forum.*

eReferences

1. Gewertz C. Testing for COVID-19 at School: Frequently Asked Questions. *Education Week*. https://www.edweek.org/leadership/should-schools-test-students-and-staff-for-covid-19/2021/03. Published March 16, 2021. Accessed January 15, 2024.
2. Home (OTC) COVID Rapid Tests. Code 1 Supply. Accessed January 16, 2024. https://www.code1supply.com/home-otc-covid-rapid-tests
3. Point Of Care Testing Products (POCT). Peach Medical Corp. Accessed January 16, 2024. https://shop.peachmedical.com/products/Point-Of-Care-Testing-Products-POCT-c129696587
4. Wholesale & Bulk COVID-19 Test Kits. WeShieldDirect.com. Accessed January 16, 2024. https://weshielddirect.com/bulk-wholesale-covid-19-test-kits/
5. INDICAID PoC (Professional) COVID-19 RAPID ANTIGEN TESTS (25 TESTS). PHASE Scientific. Accessed January 16, 2024. https://shop.indicaidusa.com/products/indicaid-covid-19-rapid-antigen-test
6. Paltiel AD, Zheng A, Sax PE. Clinical and Economic Effects of Widespread Rapid Testing to Decrease SARS-CoV-2 Transmission. *Ann Intern Med*. 2021;174(6). doi:https://doi.org/10.7326/m21-0510
7. Hurtado AV, Nguyen HT, Schenkel V, et al. The economic cost of implementing antigen-based rapid diagnostic tests for COVID-19 screening in high-risk transmission settings: evidence from Germany. *Health Econ Rev*. 2022;12(1). doi:https://doi.org/10.1186/s13561-022-00361-3
8. U.S. Bureau of Labor Statistics. Occupational Employment and Wage Statistics. Published March 31, 2022. Accessed December 31, 2022. https://www.bls.gov/oes/2021/may/naics4_623100.htm
9. Chin ET, Leidner D, Lamson L, et al. Protection against Omicron from Vaccination and Previous Infection in a Prison System. *N Engl J Med*. 2022;387(19):1770-1782. doi:https://doi.org/10.1056/nejmoa2207082
10. Chidambaram P, 2022. A Look at Vaccine and Booster Rates in Nursing Facilities as New Boosters Become Available. KFF. Published October 6, 2022. Accessed December 31, 2022. https://www.kff.org/medicaid/issue-brief/a-look-at-vaccine-and-booster-rates-in-nursing-facilities-as-new-boosters-become-available/
11. Centers for Medicare & Medicaid Services Data. COVID-19 Nursing Home Data. Accessed May 21, 2023. https://data.cms.gov/covid-19/covid-19-nursing-home-data
12. The New York Times. See How Vaccinations Are Going in Your County and State. Published October 20, 2022. Accessed May 21, 2023. https://www.nytimes.com/interactive/2020/us/covid-19-vaccine-doses.html
13. Centers for Disease Control and Prevention. COVID-19 Vaccinations in the United States. Centers for Disease Control and Prevention. Accessed December 31, 2022. https://covid.cdc.gov/covid-data-tracker/#vaccinations_vacc-people-booster-percent-pop5
14. Xin H, Wang Z, Feng S, et al. Transmission dynamics of SARS-CoV-2 Omicron variant infections in Hangzhou, Zhejiang, China, January-February 2022. *Int J Infect Dis*. 2023;126:132-135. doi:https://doi.org/10.1016/j.ijid.2022.10.033
15. Centers for Disease Control and Prevention. CDC Updates and Shortens Recommended Isolation and Quarantine Period for General Population. Published December 27, 2021. Accessed January 25, 2024. https://archive.cdc.gov/#/details?url=https://www.cdc.gov/media/releases/2021/s1227-isolation-quarantine-guidance.html
16. Baker JM, Nakayama JY, O'Hegarty M, et al. SARS-CoV-2 B.1.1.529 (Omicron) Variant Transmission Within Households — Four U.S. Jurisdictions, November 2021–February 2022. *MMWR Morb Mortal Wkly Rep*. 2022;71(9):341-346. doi:https://doi.org/10.15585/mmwr.mm7109e1
17. Ma Q, Liu J, Liu Q, et al. Global Percentage of Asymptomatic SARS-CoV-2 Infections Among the Tested Population and Individuals With Confirmed COVID-19 Diagnosis. *JAMA Netw Open*. 2021;4(12):e2137257. doi:https://doi.org/10.1001/jamanetworkopen.2021.37257
18. Joung SY, Ebinger JE, Sun N, et al. Awareness of SARS-CoV-2 Omicron Variant Infection Among Adults With Recent COVID-19 Seropositivity. *JAMA Netw Open*. 2022;5(8):e2227241. doi:https://doi.org/10.1001/jamanetworkopen.2022.27241

© 2024 Dong S et al. *JAMA Health Forum.*

19. Pollock NR, Jacobs JR, Tran K, et al. Performance and Implementation Evaluation of the Abbott BinaxNOW Rapid Antigen Test in a High-Throughput Drive-Through Community Testing Site in Massachusetts. Loeffelholz MJ, ed. *J Clin Microbiol*. 2021;59(5). doi:https://doi.org/10.1128/jcm.00083-21
20. Schrom J, Marquez C, Pilarowski G, et al. Comparison of SARS-CoV-2 Reverse Transcriptase Polymerase Chain Reaction and BinaxNOW Rapid Antigen Tests at a Community Site During an Omicron Surge. *Ann Intern Med*. 2022;175(5). doi:https://doi.org/10.7326/m22-0202
21. Dryden-Peterson S, Kim A, Kim AY, et al. Nirmatrelvir Plus Ritonavir for Early COVID-19 in a Large U.S. Health System. *Ann Intern Med*. 2022;176(1):77-84. doi:https://doi.org/10.7326/m22-2141

© 2024 Dong S et al. *JAMA Health Forum.*
