# Supplementary material for: Screening Strategies to Reduce COVID-19 Mortality in Nursing Homes
Source: JAMA Health Forum. 2024 Apr 26;5(4):e240688. doi: 10.1001/jamahealthforum.2024.0688 (PMC11065177; doi:10.1001/jamahealthforum.2024.0688)
Supplement: Supplement 2. — Data Sharing Statement [file jamahealthforum-e240688-s002.pdf]

Data Sharing Statement

Dong. Screening Strategies to Reduce COVID-19 Mortality in Nursing Homes. *JAMA Health Forum*. Published April 26, 2024. doi:10.1001/jamahealthforum.2024.0688

Data
Data available: Yes
Data types: Other (please specify)
Additional Information: All model and replication code
How to access data: https://github.com/sdong127/nursing-home
When available: With publication

Supporting Documents
Document types: Statistical/analytic code
How to access documents: https://github.com/sdong127/nursing-home
When available: With publication

Additional Information
Who can access the data: Anyone
Types of analyses: Anyone
Mechanisms of data availability: Public
